# Diagnostic Performance of Large Language Models for Orthopedic-Related Rare Diseases and Their Impact on Physicians’ Diagnostic Accuracy: 2-Stage Comparative Evaluation Study Based on the Chinese Rare Disease Catalog

**DOI:** 10.2196/92931

**Published:** 2026-07-24

**Authors:** Tusheng Li, Ziqian Ma, Baodong Wang, Ning Fan, Aobo Wang, Lei Zang

**Affiliations:** 1 Beijing Chao-Yang Hospital, Capital Medical University Beijing China

**Keywords:** clinical decision support systems, diagnostic accuracy, large language model, LLM, orthopedics, rare diseases

## Abstract

**Background:**

Orthopedic-related rare diseases are difficult to diagnose because of their low prevalence, heterogeneous phenotypes, and fragmented knowledge. Large language models (LLMs) can serve as dynamic knowledge-support tools, but their diagnostic performance and effect on physicians’ decision-making remain unclear.

**Objective:**

This study aims to compare the diagnostic performance of advanced LLMs for orthopedic-related rare diseases and to evaluate the effect of a 2-stage LLM-assisted diagnostic workflow on physicians’ diagnostic accuracy and subjective acceptance.

**Methods:**

We selected 40 orthopedic-related rare diseases from the Chinese Rare Disease Catalog. A total of 4 general-purpose LLMs each generated 1 primary diagnosis and 5 differential diagnoses per case. Diagnostic accuracy, defined as a correct primary diagnosis, was compared using the Cochran Q test and pairwise McNemar tests with Bonferroni correction. A representative LLM was integrated into a 2-stage workflow involving 27 intermediate and 15 senior orthopedic physicians. Physicians first diagnosed all cases independently and then rediagnosed the same cases after reviewing nonauthoritative LLM suggestions. Physician diagnostic data were primarily analyzed using mixed-effects logistic regression at the individual-diagnosis level. Case-level group accuracy was additionally assessed using >50% and ≥2/3 accurate-physician thresholds. After both rounds, physicians completed an 8-item Likert-scale questionnaire assessing subjective acceptance and workflow perceptions.

**Results:**

Claude Sonnet 4.5, ChatGPT-5.0, and Gemini 2.5 Pro each achieved 90% (36/40) primary-diagnosis accuracy, whereas DeepSeek-V3.2 achieved 67.5% (27/40; Cochran Q *P*<.001). Before LLM assistance, mean physician-level accuracy was 42.22% for intermediate physicians and 58.67% for senior physicians; after assistance, it increased to 68.80% and 83.33%, respectively. In the primary mixed-effects logistic regression analysis, physician seniority group and LLM assistance stage were significantly associated with diagnostic correctness (both *P*<.001), whereas the group-by-stage interaction was not significant (*P*=.10). Secondary case-level analyses using the >50% threshold showed improvement from 40% (16/40) to 67.5% (27/40) for intermediate physicians and from 57.5% (23/40) to 82.5% (33/40) for senior physicians, with similar findings using the ≥2/3 threshold. Cases accurately diagnosed by all 3 agents increased from 16 to 27. The questionnaire showed high internal consistency (Cronbach α=0.902) and generally positive attitudes, with no significant differences between physician groups (*P*=.11 to *P*=.78).

**Conclusions:**

LLMs achieved high diagnostic accuracy for orthopedic-related rare diseases. In the 2-stage LLM-assisted workflow, LLM assistance was associated with higher diagnostic correctness in both physician groups, although seniority-related differences in the magnitude of benefit require evaluation in larger studies. Senior physicians retained higher diagnostic correctness than intermediate physicians. Secondary case-level analyses suggested attenuation of group-level gaps in case-recognition patterns. Physicians reported broadly positive workflow perceptions. Given the same-day repeated-case design and potential short-term recall bias, these exploratory findings should be interpreted cautiously and warrant prospective randomized, crossover, washout-period, independent-case, or real-world evaluations of LLM-assisted diagnostic workflows in orthopedics.

## Introduction

Rare diseases have emerged as a growing research priority in public health, characterized by low prevalence, heterogeneous clinical phenotypes, fragmented diagnostic pathways, and prolonged diagnostic delays [1-3]. Although genomic testing has improved rare disease diagnosis, its overall diagnostic yield remains <50%, and cost and expertise requirements limit its widespread use [3-5]. In routine practice, frontline clinicians rarely encounter these diseases and often have limited access to structured learning resources, making missed or delayed diagnoses common in nonspecialized settings [6-9].

To improve rare disease management, China has issued 2 batches of the National Rare Disease Catalog, listing 207 diseases selected according to prevalence, severity, diagnostic clarity, and treatment availability [5]. This catalog provides an important framework for rare disease management, but a key digital medicine challenge is how to transform dispersed knowledge from guidelines, expert consensus documents, textbooks, and registries into usable decision-support tools for frontline clinicians.

Traditional clinical decision support systems (CDSSs) mainly rely on manually programmed rules or limited knowledge bases, leading to insufficient rare disease coverage and costly maintenance that may lag behind emerging evidence [10-12]. By contrast, large language models (LLMs) can integrate knowledge from large-scale text corpora and have shown promise in medical question answering, simulated consultations, and clinical reasoning [13-15]. However, most existing studies have focused on common diseases or single-specialty scenarios, and the diagnostic performance of LLMs for rare diseases remains underexplored [2,16,17]. Therefore, integrating LLMs into the management process of rare diseases holds significant clinical value [5].

In orthopedics, early studies suggest that LLMs may facilitate clinical diagnosis and decision support; however, research has predominantly addressed common orthopedic conditions, such as hip and knee osteoarthritis, lumbar degenerative diseases, and shoulder disorders [18-20]. Systematic evaluations of LLMs in rare bone diseases or musculoskeletal rare diseases remain scarce [21,22]. Rare bone diseases represent an important yet long-underestimated category of rare diseases. It has been reported that among approximately 10,000 rare diseases, about 800 are skeletal disorders [23]. Rare bone diseases account for 5% of all birth defects [24], making them a numerically large and highly heterogeneous group within the rare disease spectrum [25]. In rare disease classification, musculoskeletal-related rare diseases are also recognized as a distinct therapeutic area, and new therapies in this field have exhibited a consistent expansion trend in recent years [23,26,27].

At the specialty level, orthopedic-related rare diseases are characterized by low incidence but high involvement [23-25]. Although many conditions have metabolic, genetic, or neuromuscular etiologies, patients often initially present to orthopedic clinics with pathological fractures, severe skeletal deformities, progressive scoliosis, muscle weakness, or gait abnormalities [28-31]. Therefore, orthopedic surgeons act as “gatekeepers” in deciding whether to suspect an underlying rare disease [23,32]. Because of a lack of systematic understanding of relevant rare diseases, orthopedic physicians may attribute these cases to more common conditions, resulting in diagnostic delays and missed opportunities for timely, guideline-concordant management [6,33,34]. On this basis, the 207 diseases listed in the Chinese Rare Disease Catalog were used as a framework, and 40 orthopedic-related rare diseases were selected, guided by orthopedic practice, to construct standardized Chinese case scenarios. The goal was to evaluate the performance of LLMs and their potential benefit to orthopedic physicians in a rare disease spectrum that more closely reflects real-world orthopedic workflows.

This study adopted a sequential design to evaluate the diagnostic performance of LLMs for orthopedic-related rare diseases and their impact on physicians’ diagnostic accuracy and subjective acceptance. Specifically, we aimed to (1) evaluate the baseline diagnostic performance of mainstream general-purpose LLMs for orthopedic-related rare diseases, (2) assess whether integrating a representative LLM into a 2-stage diagnostic workflow could improve diagnostic accuracy among intermediate and senior orthopedic physicians and whether this effect differed by physician seniority, and (3) explore physicians’ subjective acceptance of the LLM-assisted workflow, thereby informing the development of human–AI collaborative workflows in orthopedics.

## Methods

### Study Design

**Figure 1:**
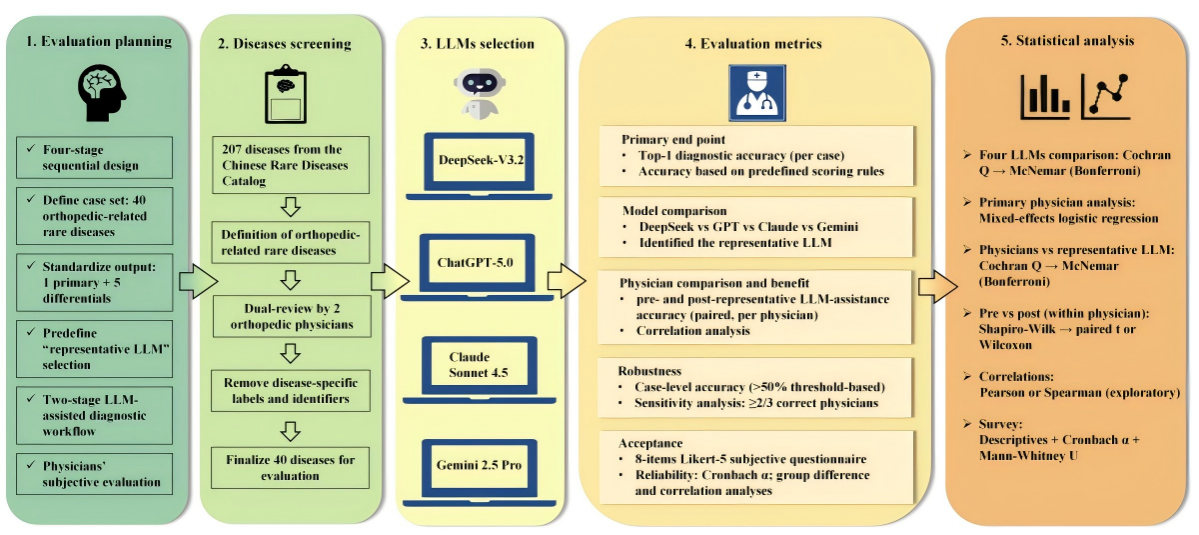
Comprehensive design flowchart of the study. LLMs: Large language models.

A 4-stage sequential design was used to evaluate LLMs in the diagnosis of orthopedic-related rare diseases and their effect on physicians’ diagnostic accuracy and subjective acceptance. First, the model evaluation stage involved 40 orthopedic-related rare diseases selected from the Chinese Rare Disease Catalog, and 4 LLMs were evaluated using the same case set to identify a representative model. Second, the baseline physician evaluation stage involved 27 intermediate and 15 senior orthopedic physicians who independently evaluated the same 40 cases and assigned initial diagnoses without LLM assistance, providing baseline diagnostic accuracy for the physicians and a comparison with the selected representative LLM. Third, the LLM-assisted evaluation stage involved physicians reviewing the representative LLM’s diagnostic suggestions, including 1 primary diagnosis and 5 differential diagnoses, and they were explicitly informed that the content might be incomplete or incorrect and required critical appraisal. The physicians then performed an LLM-assisted reassessment of the same 40 cases and assigned postassistance diagnoses. For each physician, the baseline and post-LLM–assisted diagnostic rounds were completed on the same day. After completing the independent baseline round and before completing the post-LLM–assisted round, physicians were not allowed to consult external references or search for additional disease-related information. This procedure was intended to reduce additional information-seeking and external learning effects. Pre- and post-LLM–assisted diagnostic accuracies were compared in both physician groups and contrasted with the LLM’s performance. Finally, the physician subjective evaluation stage involved physicians completing an 8-item, 5-point Likert-scale questionnaire after both rounds, assessing perceived usefulness, trust level, explainability, learning and educational value, workload and workflow fit, perceived safety, intention to use in the future, and willingness to recommend. A schematic overview of the study design is shown in Figure 1.

### Data Sources and Definition of Orthopedic-Related Rare Diseases

In 2018, China’s National Health Commission published the first batch of the Chinese Rare Disease Catalog, which included 121 rare diseases, followed by a second batch of 86 diseases in 2023. The 207 diseases were selected using the following criteria: (1) low prevalence (prevalence <1/500,000 or incidence in newborns <1/10,000), (2) considerable health impact, (3) clearly defined diagnostic protocols, and (4) availability of feasible treatment options. The clinical phenotypes for catalog diseases were obtained from the National Rare Disease Registry System (NRDRS) [35], which had integrated data from 107 collaborating institutions and 92,600 cases as of 2025.

Based on the 207 catalog diseases and the disease scope documented in *Rare Orthopedic Diseases* [36], orthopedic-related rare diseases were defined as follows: (1) rare diseases in which the bone and joint system is the main system involved, requiring direct diagnosis, treatment, or long-term management by orthopedic physicians (eg, osteogenesis imperfecta, congenital scoliosis, and osteosarcoma) or (2) rare diseases in which the primary etiology does not originate in the bone or joint system, but orthopedic problems frequently occur during the clinical course, requiring orthopedic intervention or management participation (eg, hereditary spastic paraplegia, congenital myasthenic syndrome, and congenital myotonia). Diseases were excluded if they (1) rarely involved the bones or joints and did not require orthopedic follow-up or intervention or (2) were associated only with occasional nonspecific skeletal changes and orthopedic complications were not a major clinical burden. Because the aim was to construct an orthopedic-relevant rare disease test set rather than a random or prevalence-representative sample of all cataloged rare diseases, we used a purposive, criterion-based, disease-level screening approach. Two orthopedic physicians (TL, 5 years of experience; BW, 9 years of experience) independently screened the catalog diseases using these criteria. Discrepancies were reviewed by the study expert panel, which included orthopedic physicians involved in the study design and case construction, and final decisions were made by consensus based on the predefined orthopedic relevance criteria. Ultimately, 40 orthopedic-related rare diseases were included. Each included disease contributed 1 standardized case scenario; therefore, the 40 cases corresponded to 40 distinct orthopedic-related rare diseases, and no disease was represented by more than 1 case. To mimic patient narratives during clinical encounters, deidentified symptom descriptions from the NRDRS for these diseases were systematically standardized, and disease-specific labels and identifiers were removed. The standardization process followed a prespecified template. Original NRDRS-derived symptom descriptions were converted into concise clinical vignettes that retained the main presenting symptoms, key musculoskeletal manifestations, relevant systemic or neuromuscular features, and disease course information, when available. Diagnosis-revealing information, including disease names, eponyms, genetic labels, and pathognomonic identifiers, was removed. Wording was harmonized to maintain comparable clarity and diagnostic information density, and no additional diagnostic clues or clinical information were added beyond the source description. The screening steps for orthopedic-related rare diseases are outlined in Multimedia Appendix 1.

### Selection of LLMs

Four general-purpose LLMs were compared: DeepSeek-V3.2, ChatGPT-5.0, Claude Sonnet 4.5, and Gemini 2.5 Pro. The selection was based on three considerations: (1) all 4 are representative general-purpose LLMs developed by different teams using different architectures and training strategies, reflecting the current performance level of mainstream LLMs; (2) they have demonstrated strong performance in general reasoning and medical and scientific question-answering tasks and are widely accessible, thereby facilitating future clinical validation and implementation; and (3) all models support Chinese input and output and have demonstrated good adaptability in Chinese medical and scientific tasks, aligning with our Chinese scenario design based on the Chinese Rare Disease Catalog. The main features of the 4 LLMs are summarized in Multimedia Appendix 2.

### Diagnostic Procedures

The 4 LLMs and all physicians worked from the same set of 40 Chinese cases. For the LLM evaluation, each model was prompted in Chinese: “As an orthopedic surgeon, based on the above clinical manifestations, provide one primary clinical diagnosis for this disease and five differential diagnoses.” All model queries were performed by 1 investigator (ZM, 6 years of experience) within a 20-day evaluation period using the publicly available web interface of each model. To avoid dialogue mixing, each case was queried once independently for each model in a separate single-turn session with truncated conversation history. The same Chinese prompt was used for all models, and each model was required to output 1 primary clinical diagnosis and 5 differential diagnoses for each case. Each model response was recorded. The default web interface settings were used, and no generation parameters were manually adjusted. The runtime conditions and prompt-control settings are summarized in Multimedia Appendix 2. A total of 2 clinicians independently reviewed the LLM outputs while blinded to model identity. In case of disagreement, a third physician, who was also blinded to model identity, was consulted to reach consensus through in-depth discussion. Similarly, physician diagnostic outputs were anonymized before grading, and the evaluators were blinded to the participating physicians’ identities and seniority levels when determining diagnostic correctness. This procedure was used to reduce potential leniency bias related to physician status or professional title, particularly when judging synonymous or clinically equivalent diagnostic terms.

In this study, the “accurate diagnosis” of a case was defined as follows: when the primary diagnosis aligned with the reference standard answer, it was recorded as an accurate diagnosis. The 5 differential diagnoses were excluded from the assessment of the primary outcome (accuracy rate). This end point was selected because it provided a predefined binary outcome that could be consistently applied to both LLMs and physicians and was suitable for the planned comparative analyses. If uncertainty or multiple candidate diagnoses were expressed, only the explicitly stated final primary diagnosis was used to determine diagnostic correctness. As a general principle, if the model or physician used a clinical term unambiguously referring to the same disease entity, the diagnosis was counted as correct. Nevertheless, during the model evaluation and subsequent LLM-assisted physician support phases, the 5 differential diagnoses provided in the model output were retained for 2 reasons. First, this more closely resembles real clinical reasoning, in which physicians typically formulate a primary diagnosis alongside a ranked list of differential diagnoses. Preservation of this structure enabled the simulation of initial and differential diagnostic reasoning rather than a single-label classification. Second, in the LLM-assisted stage, physicians were presented with a set of information comprising a primary diagnosis and several candidate differential diagnoses. After reviewing the diagnoses and brief rationale provided by the LLM, they critically evaluated this information and integrated it into their own knowledge to make an independent judgment, reinforcing the role of the LLM as an auxiliary source of decision-making information rather than a replacement for human diagnosis.

As the primary end point was strictly defined as “primary diagnosis accurate vs inaccurate,” and differential diagnoses were excluded from the accuracy metric, physicians were not required to record full differential lists in either round. For each case, intermediate and senior physicians provided only a single final primary diagnosis. This design was primarily chosen for two main reasons: (1) the main study questions focused on primary diagnostic accuracy and its change with LLM assistance, and collecting large numbers of differential diagnoses would not directly serve the primary end point; and (2) the diagnostic task already involved 40 cases completed by 42 physicians across 2 rounds, which represented a considerable study-related response burden. Requiring comprehensive differential diagnosis lists in both rounds would have further increased the experimental workload and potentially reduced response quality and compliance.

Throughout the study, LLMs were considered decision-support tools rather than autonomous diagnosticians. After the first independent round, the physicians could view the selected representative LLM’s output (1 primary diagnosis and 5 differential diagnoses), mimicking a “checking reference materials and consulting an expert” information environment. Before the second round, it was clearly emphasized that the LLM’s suggestions might be incomplete or incorrect and that physicians must rely on clinical information and their own expertise to make the final diagnosis. During the interval between the 2 rounds, physicians were instructed to review only the representative LLM output provided by the study team and were not permitted to consult textbooks, guidelines, online resources, or other external reference materials. By comparing pre- and post-LLM–assisted diagnostic accuracies in both physician groups and contrasting these with the LLM’s standalone performance, the study aimed to determine whether LLMs can improve overall diagnostic performance and whether the effect of LLM assistance differs by physician seniority, without undermining physician autonomy.

Notably, 2 diseases required specific handling due to differences between catalog names and the clinical descriptions used to construct the cases. First, generalized myasthenia gravis: the catalog name is “generalized myasthenia gravis,” but in the section referenced for constructing this case, the clinical presentation uses the description “myasthenia gravis.” Therefore, “myasthenia gravis” and “generalized myasthenia gravis” were considered to be correct. Second, spinocerebellar ataxia (SCA): the catalog entry is “spinocerebellar ataxia,” whereas the clinical presentation drew on a section describing SCA3. Accordingly, “SCA” and “SCA3” were considered to be correct. Apart from these 2 predefined exceptions, overly broad superordinate labels (eg, diagnosing “primary hereditary dystonia” as simply “dystonia”) and overly narrow subtypes (eg, diagnosing “SCA” as “hereditary SCA”) or mismatched disease names were scored as incorrect.

In this study, an LLM was considered the representative model if its primary diagnosis accuracy was higher than that of the other LLMs. When multiple LLMs achieved equally high performance and were tied in strict primary-diagnosis accuracy, they were treated as a subset of high-performing general-purpose LLMs with comparable diagnostic capability. However, because the subsequent 2-stage physician–LLM-assisted diagnostic workflow required the inclusion of only 1 LLM to avoid an overly complex study design and to reduce the intervention burden on participating physicians, we prespecified in the protocol that 1 representative model would be selected from this high-performing subset as the reference LLM for the subsequent workflow.

To conduct this secondary selection, inspired by the catalog-driven research approach proposed by Zhong et al [5], we additionally counted, among models tied in strict primary-diagnosis accuracy, the number of cases in which the target disease was correctly captured at the level of the primary diagnosis plus 5 differential diagnoses. Based on this secondary quantitative assessment, we ultimately determined a single representative LLM for the subsequent 2-stage workflow.

### Physician Selection

The physician cohort selected for this study mainly comprised intermediate and senior orthopedic physicians, excluding junior physicians, primarily for reasons of clinical representativeness and study aims. In real-world practice, orthopedic-related rare diseases are encountered and predominantly managed by intermediate and senior doctors, who typically lead diagnostic and treatment decisions in specialized outpatient clinics and inpatient settings. The aim of the study was to evaluate the performance of the LLM compared with that of clinicians who are actually responsible for rare disease diagnosis and management and to measure the added value of LLM support in this group. In total, 27 intermediate and 15 senior orthopedic physicians were enrolled. Physicians were classified according to their professional titles in the Chinese hospital system. Intermediate physicians were defined as attending orthopedic physicians, whereas senior physicians were defined as associate chief or chief orthopedic physicians. Professional title was used as the primary grouping criterion because it reflects clinical experience, institutional credentialing, and responsibility for diagnostic decision-making in routine Chinese orthopedic practice. Baseline demographic and professional characteristics of the participating physicians, including years in orthopedic practice, gender, orthopedic subspecialty, and practice setting, are provided in Multimedia Appendix 3. The diverse group of physicians reflects the typical heterogeneity of diagnostic experience in real-world orthopedic practice. This sample size improved the robustness of exploratory comparisons among intermediate physicians, senior physicians, and the LLM and allowed analysis of changes in pre- and post-LLM–assisted diagnostic accuracies among physicians, although the study was not primarily powered to detect interaction effects between physician seniority and LLM assistance stage. Furthermore, it reduced the impact of individual physicians’ outlier performance on the experiment.

### Physician Subjective Questionnaire Evaluation

After completing both diagnostic rounds, all physicians filled out a brief subjective questionnaire. The instrument consisted of 8 items covering perceived usefulness, trust level, explainability, learning and educational value, workload and workflow fit, perceived safety, intention to use in the future, and willingness to recommend. Each item was rated on a 5-point Likert scale (1=strongly disagree, 2=disagree, 3=neutral, 4=agree, and 5=strongly agree). The questionnaire was developed by the study team using core constructs from the technology acceptance model (TAM), the unified theory of acceptance and use of technology (UTAUT), the Trust in Automation Scale, and prior research on the acceptance of CDSSs and AI tools [37-43]. The main objectives were to describe orthopedic physicians’ subjective experience and acceptance of the LLM-assisted diagnostic workflow and to supplement objective diagnostic metrics. Item wording is provided in Multimedia Appendix 4.

### Statistical Analysis

All primary statistical analyses were conducted using SPSS (version 25.0; IBM Corp). The diagnostic accuracy rate was defined as the proportion of accurate diagnoses among the 40 cases of orthopedic-related rare diseases.

Comparison of LLM diagnostic performance: using the same 40 cases, the diagnostic performance of the 4 LLMs (DeepSeek-V3.2, ChatGPT-5.0, Claude Sonnet 4.5, and Gemini 2.5 Pro) was first compared. The Cochran Q test was used to compare differences in overall accuracy among the 4 models. If the Cochran Q was significant (*P*<.05), pairwise McNemar tests were conducted, with Bonferroni correction for 6 pairwise comparisons (adjusted significance threshold: .05/6).

For the physician diagnostic data, the primary inferential analysis was performed at the level of individual diagnostic attempts. Each observation represented 1 physician’s diagnosis of 1 case at 1 diagnostic stage, with diagnostic correctness coded as a binary outcome. A mixed-effects logistic regression model was fitted with diagnostic correctness as the dependent variable. LLM assistance stage, physician seniority group, and their interaction were included as fixed effects. To account for the hierarchical and paired structure of the data, random intercepts were specified for physician identity, case identity, and the physician-by-case pair. The physician-level random intercept accounted for clustering of repeated diagnoses within the same physician, the case-level random intercept accounted for variation in case difficulty, and the physician-by-case random intercept accounted for the paired dependency between the diagnoses made before and after LLM assistance by the same physician for the same case. A binomial distribution with a logit link function was used. Fixed-effect tests were reported using *F* test statistics and *P* values using the SPSS GENLINMIXED procedure. An equivalent generalized linear mixed-effects model with the same fixed-effect terms and random-intercept structure was fitted in R (version 4.5.1; R Core Team) using the *glmmTMB* package (version 1.1.14) to report fixed-effect coefficient estimates and random-effect variance estimates with SEs. The R model included fixed effects for LLM assistance stage, physician seniority group, and their interaction, and random intercepts for physician identity, case identity, and the physician-by-case pair. The interaction term was used to examine whether the effect of LLM assistance differed between intermediate and senior physicians.

To facilitate comparison with the original group-level summaries and to examine the robustness of the findings, case-level majority-threshold analyses were retained as secondary sensitivity analyses. For each disease, the number of physicians in a group who answered correctly was counted. If more than 50% of physicians in that group answered correctly, the case was labeled as accurately diagnosed by that group. A stricter threshold of ≥2/3 accurate physicians was further applied as a sensitivity analysis. These case-level analyses were used to describe group-level diagnostic consistency patterns and were not treated as the primary inferential analysis. This yielded 3 paired binary categorical data series (intermediate, senior, and representative LLM groups) for the pre- and post-LLM–assisted diagnostic time points. At each time point, the Cochran Q test was used to compare case-level overall accuracy among the 3 groups. If Cochran Q was significant (*P*<.05), pairwise McNemar tests were conducted with Bonferroni correction for 3 comparisons (adjusted significance threshold: .05/3).

Physician-level effect of LLM assistance was evaluated to determine the effect of the representative LLM’s assistance at the individual level. Each physician’s accuracy before and after LLM assistance was calculated across the 40 cases, yielding 27 and 15 paired observations in the intermediate and senior groups, respectively. The Shapiro-Wilk test was used to examine the normality of the differences in accuracy rates before and after LLM assistance. When the differences were found to have a normal distribution, 2-tailed paired *t* tests were used to compare the mean accuracy rates before and after LLM assistance; otherwise, the Wilcoxon signed-rank test was applied. As an exploratory analysis, whether physicians with lower baseline accuracy benefited more from LLM assistance was also determined. Pearson correlation was used to evaluate the linear relationship between baseline accuracy and the magnitude of accuracy improvement within the intermediate and senior groups separately, reporting correlation coefficients (*r*) and *P* values.

As an exploratory descriptive analysis, diagnostic transition patterns before and after LLM assistance were summarized at the physician-case pair level. Transitions were classified as accurate before and after assistance, changed from accurate to inaccurate, changed from inaccurate to accurate, or inaccurate before and after assistance and were reported as counts and percentages.

Physicians’ subjective evaluation was analyzed using the following methods. For the 8 questionnaire items, descriptive statistics (mean [SD] and median [IQR]) were calculated. The internal consistency of the 8 items was evaluated using Cronbach α. To compare subjective evaluations between intermediate and senior physicians, the Mann-Whitney *U* test was used for the overall mean score (average of the 8 items) and for each item. Finally, as an exploratory analysis, the association between subjective acceptance and objective benefit was examined. The overall mean questionnaire score was used as an indicator of subjective acceptance of the LLM-assisted diagnostic tool, whereas accuracy improvement was used as an indicator of objective benefit. Spearman rank correlation was used to assess the association between these 2 variables.

### Ethical Considerations

This study was reviewed and approved by the Ethics Committee of Beijing Chaoyang Hospital, Capital Medical University (approval number 2025-KE-417). Ethical approval was obtained before the initiation of LLM querying, physician diagnostic evaluation, and questionnaire data collection. The study used standardized simulated cases and did not involve real patient interventions or identifiable patient information. The participating physicians completed simulated diagnostic tasks and a subjective questionnaire. The ethics committee approved a waiver of written informed consent. Participation was voluntary, and all physician-level data were anonymized before analysis and reported only in aggregate. No personally identifiable information about the participating physicians was included in the manuscript or supplementary materials. No identifiable images of participants were used. The participating physicians received no financial compensation for participation.

## Results

### Diagnostic Performance of the 4 LLMs

This study included the 40 orthopedic-related rare diseases and used 4 models for diagnostic performance evaluation. Accuracy was 67.5% (27/40) for DeepSeek-V3.2, 90% (36/40) for ChatGPT-5.0, 90% (36/40) for Claude Sonnet 4.5, and 90% (36/40) for Gemini 2.5 Pro (Table 1). The case questionnaire and LLM evaluation results for 40 orthopedic-related rare diseases are presented in Multimedia Appendices 5 and 6, respectively.

**Figure 2:**
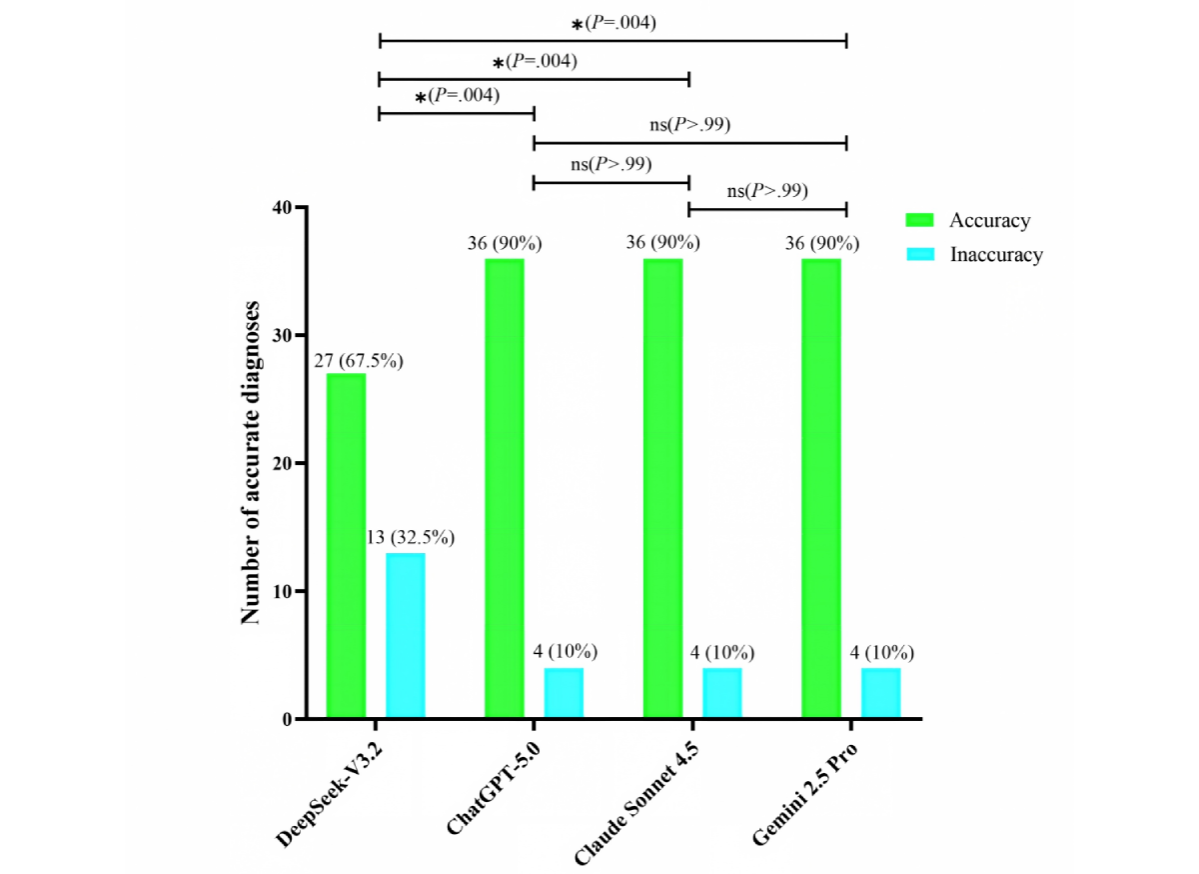
Schematic diagram comparing 4 large language models. *P<.05/6; "ns" indicates a nonsignificant difference.

The Cochran Q test indicated significant overall differences among the 4 models (*P*<.001). Pairwise McNemar tests showed that DeepSeek-V3.2 performed significantly worse than ChatGPT-5.0, Claude Sonnet 4.5, and Gemini 2.5 Pro (all *P*=.004, <.05/6), whereas no significant differences were observed among ChatGPT-5.0, Claude Sonnet 4.5, and Gemini 2.5 Pro (all *P*>.99, >.05/6; Figure 2).

Given that the 3 LLMs exhibited identical primary diagnostic accuracy rates, we further examined which target diseases were accurately identified either as the primary diagnosis or among the 5 differential diagnoses. The results indicated that in rare disease cases, in which the primary diagnosis was judged inaccurate, Claude Sonnet 4.5 still included accurate diagnoses within the differential diagnosis list for some cases (eg, “asphyxiating thoracic dystrophy,” for which the primary diagnosis was inaccurate but the differential diagnosis list contained the accurate diagnosis), whereas ChatGPT-5.0 and Gemini 2.5 Pro did not list the accurate diagnosis in their differential diagnoses. Consequently, based on this secondary quantitative assessment, Claude Sonnet 4.5 was selected as the representative high-performing LLM for the 2-stage workflow.

**Table 1:** Diagnostic performance results for 4 large language models (LLMs).

LLM	Accuracy, n (%)	Inaccuracy, n (%)
DeepSeek-V3.2	27 (67.5)	13 (32.5)
ChatGPT-5.0	36 (90)	4 (10)
Claude Sonnet 4.5	36 (90)	4 (10)
Gemini 2.5 Pro	36 (90)	4 (10)

### Baseline and Postassistance Diagnostic Performance of Physicians

The baseline demographic and professional characteristics of the 42 orthopedic physicians are summarized in Multimedia Appendix 3. A total of 27 intermediate and 15 senior orthopedic physicians completed 2 rounds of diagnoses for the same 40 cases (the physicians’ diagnostic findings are presented in Multimedia Appendix 7).

Before LLM assistance, the mean physician-level accuracy rates were 42.22% and 58.67% for intermediate and senior physicians, respectively. After reviewing the suggestions of Claude Sonnet 4.5, the mean physician-level accuracy rates increased to 68.80% and 83.33% for intermediate and senior physicians, respectively. These descriptive results showed higher diagnostic accuracy in both physician groups after LLM assistance.

### Primary Mixed-Effects Logistic Regression Analysis

In the primary mixed-effects logistic regression analysis including all 3360 individual diagnostic attempts, random intercepts were specified for physician identity, case identity, and the physician-by-case pair. Both physician seniority group and LLM assistance stage were significantly associated with diagnostic correctness (Table 2). An equivalent R generalized linear mixed-effects model yielded the same qualitative fixed-effect pattern, with physician seniority group and LLM assistance stage remaining statistically significant and the group-by-stage interaction remaining nonsignificant. The complete R model results, including fixed-effect estimates and random-effect variance estimates with SEs, are provided in Multimedia Appendix 8.

Diagnostic correctness differed significantly between intermediate and senior physicians (*F*_1,3356_=45.335; *P*<.001) and between the pre- and post-LLM assistance stages (*F*_1,3356_=317.775; *P*<.001). The interaction between physician seniority group and LLM assistance stage did not reach statistical significance (*F*_1,3356_=2.762; *P*=.10). Therefore, the model did not provide conclusive evidence that the effect of LLM assistance differed by physician seniority. However, given the relatively small physician sample, a potential seniority-related difference in the magnitude of benefit cannot be excluded.

These findings indicate that LLM assistance was significantly associated with higher diagnostic correctness after accounting for repeated diagnoses by the same physician, variation in case difficulty, and paired dependency at the physician-case level. Senior physicians also showed higher overall diagnostic correctness than intermediate physicians. However, the nonsignificant interaction should not be interpreted as evidence of equivalent benefit across the 2 physician groups; larger studies are needed to determine whether physician seniority modifies the effect of LLM assistance.

**Table 2:** Primary mixed-effects logistic regression analysis of diagnostic correctness using all individual diagnostic attempts.

Model component	Parameter	Variance estimate	SE	*F* test (*df*)	*P* value
Fixed effect^a^	Physician seniority group	—^b^	—	45.335 (1, 3356)	<.001
Fixed effect^a^	LLM^c^ assistance stage	—	—	317.775 (1, 3356)	<.001
Fixed effect^a^	Physician seniority group × LLM assistance stage	—	—	2.762 (1, 3356)	.10
Random effect^d^	Physician identity random intercept	7.908	4.242	—	—
Random effect	Case identity random intercept	516.644	159.534	—	—
Random effect	Physician-by-case pair random intercept	343.769	72.585	—	—

^a^Fixed-effect tests were obtained from the primary SPSS analysis.

^b^Not applicable.

^c^LLM: large language model.

^d^Random-effect variance estimates and SEs were obtained from an equivalent generalized linear mixed-effects model fitted in R (version 4.5.1) using the *glmmTMB* package (version 1.1.14) with the same fixed-effect terms and random-intercept structure.

### Secondary Case-Level Majority-Threshold Analyses

We further performed secondary case-level analyses using the >50% and ≥2/3 majority thresholds. At the case level, using the >50% threshold before LLM assistance, the accuracy rate was 40% (16/40) for the intermediate group, 57.5% (23/40) for the senior group, and 90% (36/40) for Claude Sonnet 4.5. The Cochran Q test revealed significant differences among the 3 groups (*P*<.001). Pairwise McNemar tests indicated significant differences for all 3 comparisons (intermediate vs senior: *P*=.016, <.05/3; intermediate vs Claude: *P*<.001, <.05/3; senior vs Claude: *P*<.001, <.05/3).

**Figure 3:**
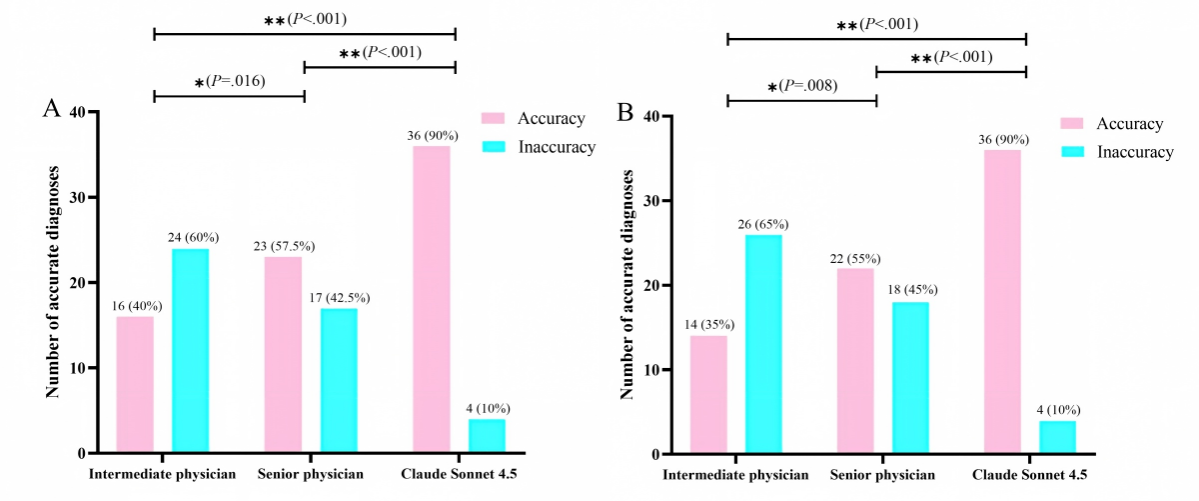
Schematic diagram of the comparison between physicians and Claude Sonnet 4.5 before large language model assistance, based on 2 criteria: (A) a >50% threshold and (B) the stricter ≥2/3 threshold. *P<.05/3, **P<.001.

Using the stricter ≥2/3 threshold before LLM assistance, the number of accurately diagnosed cases were 14 (35%), 22 (55%), and 36 (90%) for the intermediate group, senior group, and Claude Sonnet 4.5, respectively. Again, pairwise comparisons revealed that both physician groups showed significantly poorer performance than the LLM and that senior physicians outperformed intermediate physicians (intermediate vs senior: *P*=.008, <.05/3; intermediate vs Claude: *P*<.001, <.05/3; senior vs Claude: *P*<.001, <.05/3). The comparative results between the physicians and Claude Sonnet 4.5 before LLM assistance are shown in Table 3 and Figure 3.

After LLM assistance, using the >50% threshold, the accuracy rate rose to 67.5% (27/40) for the intermediate group and 82.5% (33/40) for the senior group, whereas that of Claude Sonnet 4.5 remained at 90% (36/40). The Cochran Q test showed significant differences among the 3 groups (*P*=.002). Pairwise McNemar tests revealed a significant difference between the intermediate group and Claude Sonnet 4.5 (*P*=.004, <.05/3), whereas the differences between the intermediate and senior physicians (*P*=.03, >.05/3) and between senior physicians and Claude Sonnet 4.5 (*P*=.38, >.05/3) were not significant.

**Figure 4:**
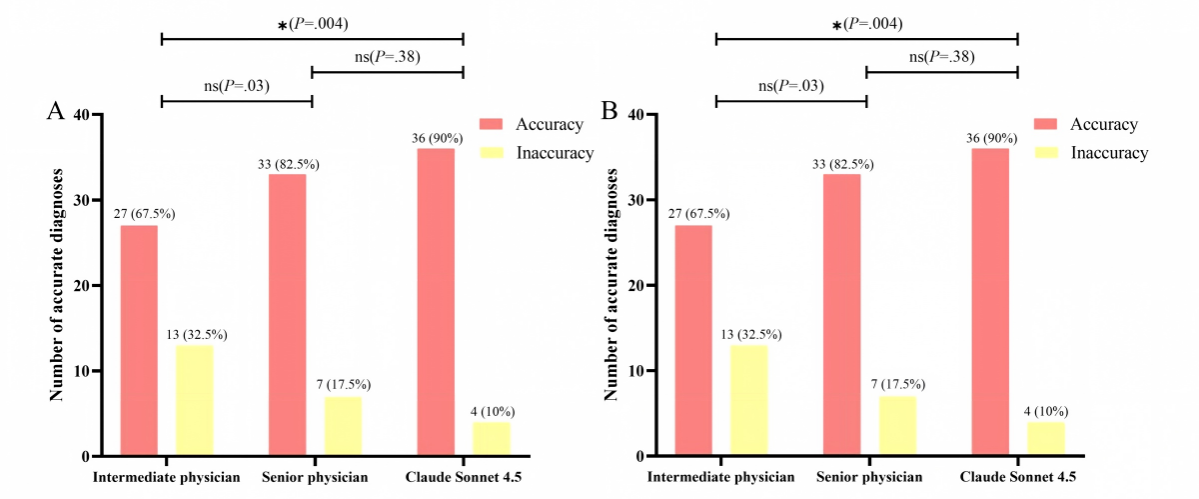
Schematic diagram of the comparison between physicians and Claude Sonnet 4.5 after large language model assistance, based on 2 criteria: (A) a >50% threshold and (B) the stricter ≥2/3 threshold. *P<.05/3; "ns" indicates a nonsignificant difference.

With the ≥2/3 threshold after LLM assistance, the results were similar: intermediate vs Claude remained significantly different, whereas intermediate vs senior and senior vs Claude were not statistically significant (intermediate vs Claude: *P*=.004, <.05/3; intermediate vs senior: *P*=.03, >.05/3; senior vs Claude: *P*=.38, >.05/3). The comparative results between the physicians and Claude Sonnet 4.5 after LLM assistance are shown in Table 4 and Figure 4.

Overall, these secondary threshold-based analyses showed a pattern broadly consistent with the primary mixed-effects model, demonstrating improved diagnostic performance after LLM assistance in both physician groups. However, because the threshold-based approach aggregated multiple individual physician diagnoses into a single case-level group outcome, it was interpreted as a descriptive sensitivity analysis rather than the primary inferential analysis. Taken together, diagnostic performance was higher in both physician groups after LLM assistance. At the group-level majority-threshold level, the gap between intermediate and senior physicians was attenuated after LLM assistance, although the primary mixed-effects model still showed a significant overall difference by physician seniority. After LLM assistance, the diagnostic accuracy of senior physicians approached that of the LLM in the secondary case-level threshold analyses.

**Table 3:** Comparison results between physicians and Claude Sonnet 4.5 before large language model (LLM) assistance.

Group	Using the >50% threshold, n (%)			Using the ≥2/3 threshold, n (%)	
	Accuracy	Inaccuracy	Accuracy		Inaccuracy
Intermediate physician	16 (40)	24 (60)	14 (35)		26 (65)
Senior physician	23 (57.5)	17 (42.5)	22 (55)		18 (45)
Claude Sonnet 4.5	36 (90)	4 (10)	36 (90)		4 (10)

**Table 4:** Comparison results between physicians and Claude Sonnet 4.5 after large language model (LLM) assistance.

Group	Using the >50% threshold, n (%)		Using the ≥2/3 threshold, n (%)	
	Accuracy	Inaccuracy	Accuracy	Inaccuracy
Intermediate physician	27 (67.5)	13 (32.5)	27 (67.5)	13 (32.5)
Senior physician	33 (82.5)	7 (17.5)	33 (82.5)	7 (17.5)
Claude Sonnet 4.5	36 (90)	4 (10)	36 (90)	4 (10)

### Effect of LLM Assistance at the Individual Physician Level

**Figure 5:**
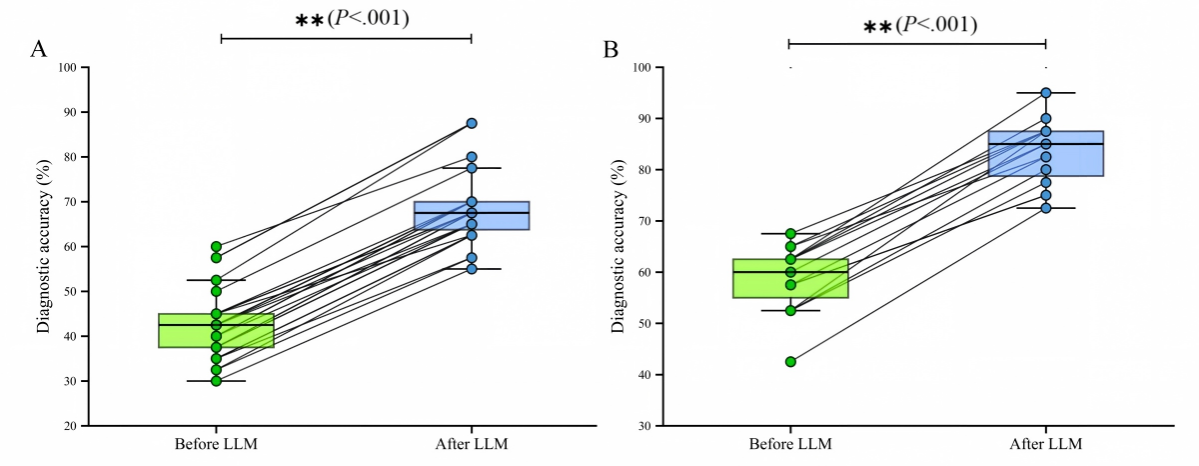
Paired box plots illustrating changes in diagnostic accuracy rate before and after representative large language model (LLM; Claude Sonnet 4.5) assistance for (A) intermediate physicians and (B) senior physicians. **P<.001.

For intermediate physicians, the mean accuracy rate increased by 26.6% (from 42.22% to 68.80%) with LLM assistance, and a statistically significant difference was observed between pre- and post-LLM assistance (paired *t* test: *P*<.001). For senior physicians, the mean accuracy rate increased by 24.7% (from 58.67% to 83.33%), with a significant difference observed between pre- and post-LLM assistance (paired *t* test: *P*<.001). Detailed physician-level diagnostic performance before and after LLM assistance is provided in Table S1 in Multimedia Appendix 9. At the individual level, all intermediate and senior physicians experienced some degree of accuracy improvement after LLM assistance, with individual gains ranging from 17.5% to 35.0%. Some intermediate practitioners with lower baseline accuracy reached or approached the accuracy levels of certain senior physicians after LLM assistance (Tables S2 and S3 in Multimedia Appendix 9; Figure 5). These descriptive findings were consistent with the primary mixed-effects analysis, which showed a significant main effect of LLM assistance stage.

However, because the mixed-effects model still demonstrated a significant overall effect of physician seniority group, the postassistance improvement should be interpreted as evidence of increased diagnostic performance in both groups rather than as elimination of experience-related differences.

When stratifying by accuracy thresholds, the number of intermediate physicians who achieved ≥50% accuracy increased from 5/27 at baseline to 27/27 after assistance; ≥60% from 1/27 to 24/27; ≥70% from 0/27 to 11/27; and ≥80% from 0/27 to 4/27 (Table S2 in Multimedia Appendix 9). None of the senior physicians achieved ≥70% accuracy before LLM assistance; after assistance, 15/15 reached ≥70%; 11/15 reached ≥80%; and 8/15 reached ≥85% (Table S3 in Multimedia Appendix 9). Pearson correlation showed almost no association between baseline accuracy and accuracy improvement among intermediate physicians (*r*=−0.009; *P*=.96), indicating that intermediate physicians with different baseline levels benefited relatively evenly from LLM assistance. Among senior physicians, baseline accuracy and accuracy improvement showed a moderate negative correlation numerically (*r*=−0.449; *P*=.09), but this association was not statistically significant and should be interpreted descriptively.

### Diagnostic Transition Patterns Before and After LLM Assistance

To further characterize how physicians’ diagnostic judgments changed after LLM assistance, we performed a physician-case–level diagnostic transition analysis (Table S4 in Multimedia Appendix 9). Among all 1680 paired diagnostic attempts, 48.1% (808/1680) were accurate both before and after LLM assistance, 25.9% (435/1680) changed from inaccurate to accurate, 26.0% (437/1680) remained inaccurate before and after assistance, and none changed from accurate to inaccurate. Among intermediate physicians, 287 of 1080 paired attempts changed from inaccurate to accurate, whereas no transition from accurate to inaccurate was observed. Among senior physicians, 148 of 600 paired attempts changed from inaccurate to accurate, and again no transition from accurate to inaccurate was observed. These findings indicate that the net accuracy improvement was mainly driven by the correction of initially inaccurate diagnoses, with no detectable deterioration of initially accurate diagnoses at the diagnostic-correctness level.

### Case-Level Patterns of Human–LLM Consistency

Using the >50% threshold, the case-level consistency patterns among intermediate physicians, senior physicians, and Claude Sonnet 4.5 were further examined.

Before LLM assistance, among the 40 cases, 16 were accurately diagnosed by all 3 groups; 13 cases were accurately diagnosed only by the LLM, whereas the diagnoses of both physician groups were mostly inaccurate; 7 cases were accurately diagnosed by the senior physician group and the LLM but not by the intermediate physician group; and 4 cases were misdiagnosed by all 3 groups (ie, a shared blind spot between humans and the LLM). After LLM assistance, the number of cases accurately diagnosed by all 3 groups increased from 16 to 27; cases accurately diagnosed only by the LLM decreased from 13 to 4; cases accurately diagnosed by the senior physician group and the LLM but not by the intermediate physician group decreased from 7 to 5; 1 additional case, asphyxiating thoracic dystrophy, was accurately diagnosed by the senior physician group but not by the intermediate physician group or the LLM; and shared blind spots (all 3 groups inaccurate) slightly decreased from 4 to 3. The specific shared blind-spot cases before and after LLM assistance are listed in Multimedia Appendix 10. With LLM assistance, 11 cases in the intermediate physician group and 10 in the senior physician group shifted from “mostly inaccurate” to “mostly accurate” at the group level. In addition, although the number of cases exclusively solved by the LLM markedly decreased, a small subset of cases remained accurately diagnosed only by the LLM, and a few remained difficult for both humans and the model, highlighting certain “weak points” that persist within the combined human–LLM system.

### Physicians’ Subjective Evaluation of the LLM-Assisted Workflow

All physicians completed a subjective questionnaire after 2 rounds of diagnosis. The questionnaire exhibited high internal consistency in this sample (Cronbach α=0.902). Given its exploratory design, the questionnaire results were interpreted descriptively as physicians’ subjective perceptions of the LLM-assisted diagnostic tool. The score distributions for each item for intermediate and senior physicians are presented in Multimedia Appendix 4. Overall, most of the items had mean scores in the “agree” to “strongly agree” range, suggesting generally positive attitudes. Dimensions related to learning and educational value, perceived safety, workload and workflow fit, as well as willingness to recommend, achieved the highest mean scores (Table S5 in Multimedia Appendix 9). These findings suggest that the physicians agreed that the LLM supplemented their knowledge and diagnostic reasoning regarding orthopedic-related rare diseases, felt that the tool was reasonably safe as long as they maintained final responsibility for the decisions, did not report a substantial additional workload when using the LLM within this simplified study workflow, and expressed a relatively high willingness to recommend similar tools to colleagues.

Comparisons between intermediate and senior physicians revealed no significant differences in overall mean questionnaire scores (Mann-Whitney *U* test *P*=.36) or in any individual item (item-level *P* values ranged from .11 to .78), indicating broadly similar acceptance across experience levels, with no strong pattern of “senior physicians being more resistant” or “intermediate physicians being more dependent” on the tool (Table S5 in Multimedia Appendix 9). As an exploratory analysis, the association between subjective acceptance and objective benefit was examined. The overall mean questionnaire score was used as an index of subjective acceptance, and accuracy improvement as an index of objective benefit. Spearman correlation revealed a very weak, nonsignificant association between the 2 (*r*=−0.058; *P*=.71), indicating that, in this sample, physicians’ perceived usefulness and willingness to use the tool were not strongly consistent with the magnitude of their actual accuracy gains.

## Discussion

### Principal Findings

Using the Chinese Rare Disease Catalog as a framework, this study constructed 40 standardized cases of orthopedic-related rare diseases and evaluated both standalone LLM diagnostic performance and a 2-stage LLM-assisted physician workflow. Overall, the evaluated mainstream LLMs showed high diagnostic accuracy in this challenging setting, and the representative model outperformed both physician groups at baseline. This suggests that general-purpose LLMs may capture clinically useful rare disease knowledge that is not consistently available to nonspecialist orthopedic physicians in routine practice.

The primary mixed-effects logistic regression analysis showed that LLM assistance was significantly associated with higher diagnostic correctness after accounting for clustering by physician, case, and physician-case pair. Senior physicians retained higher overall diagnostic correctness than intermediate physicians, whereas the group-by-stage interaction was not statistically significant. Therefore, LLM assistance improved performance in both groups, but the current data do not establish whether the magnitude of the benefit differs by physician seniority.

Secondary case-level analyses complemented the individual-level model. After LLM assistance, more cases were accurately diagnosed by all 3 agents, and fewer cases remained LLM-only or shared human–LLM blind spots. These findings suggest that LLM assistance may transform some LLM-only correct diagnoses into shared human–LLM recognition, although some difficult cases persisted. Diagnostic transition analysis further showed that improvement was mainly driven by inaccurate-to-accurate changes, with no accurate-to-inaccurate transitions observed at the diagnostic-correctness level. However, because physician confidence, reasoning processes, and reasons for accepting or rejecting LLM outputs were not recorded, these transitions should be interpreted as exploratory behavioral signals rather than definitive evidence regarding automation bias or algorithmic aversion.

Physicians reported broadly positive perceptions of the LLM-assisted workflow, particularly for learning and educational value, perceived safety, workload and workflow fit, and willingness to recommend. The weak association between subjective acceptance and objective accuracy gain suggests that perceived usefulness and actual benefit may not fully align, highlighting the need for standardized training and workflow protocols. Finally, because the same cases were used in both rounds on the same day, short-term recall bias may have contributed to the observed improvement. Thus, the findings should be interpreted as exploratory proof-of-concept evidence rather than definitive causal evidence of the absolute benefit of LLM assistance.

### Limitations

This study has several limitations. First, it used standardized cases rather than real-world clinical trials and thus did not capture contextual factors, such as imaging review or patient communication. This limitation is particularly important in orthopedics, where diagnosis typically relies on multimodal information, including imaging findings, physical examination, functional assessment, laboratory or genetic testing, and longitudinal observation. In this study, the cases were constructed from text-based registry descriptions because the Chinese Rare Disease Catalog and related registry-derived descriptions generally do not provide standardized imaging materials for most cataloged diseases. Although this text-only, catalog-driven design is consistent with prior rare disease LLM evaluations, such as the study by Zhong et al [5], it inevitably reduces ecological validity for orthopedic diagnosis. Because LLMs are primarily text-processing systems, whereas orthopedic physicians usually rely on multimodal clinical information, this vignette format may have partly favored the LLMs and may partially explain the lower baseline performance of physicians. Therefore, the baseline comparison should be interpreted as performance under a constrained text-only setting rather than as a direct reflection of real-world orthopedic diagnostic ability. Therefore, our findings reflect human–AI diagnostic performance under relatively idealized information conditions, and generalizability to routine practice requires further validation. Moreover, although the case vignettes were standardized using a prespecified template, transforming registry-derived symptom descriptions into simulated case narratives may still have introduced information loss or wording-related bias. Furthermore, no external validation dataset or real-world clinical cohort was used; therefore, the findings should be interpreted as internally controlled results from standardized simulated scenarios rather than evidence of effectiveness in routine clinical practice.

Second, it included 40 orthopedic-related rare diseases selected from the Chinese Rare Disease Catalog, covering a range of genetic bone diseases, neuromuscular conditions, and bone tumors. However, the number of diseases and cases remains limited, and the scope is limited to the Chinese-language context and orthopedics. Although the 40 diseases were selected using predefined orthopedic relevance criteria and independent screening by 2 orthopedic physicians, the selection was purposive rather than random. This approach was appropriate for constructing an orthopedic-relevant test set but was not intended to produce a prevalence-representative sample of all rare diseases in the catalog. Therefore, selection bias cannot be fully excluded. Moreover, spectrum bias may have been introduced because the standardized cases may not fully capture the broad phenotypic variability, atypical presentations, and incomplete clinical information encountered in real-world rare disease diagnosis. The included diseases and standardized case descriptions may have overrepresented relatively recognizable or textbook-like presentations rather than ambiguous real-world cases with incomplete, overlapping, or atypical findings, which may have contributed to the ceiling effect observed in some LLM performances. Therefore, whether the performance of LLMs and physicians can be extrapolated to other countries and language contexts, nonorthopedic rare diseases, and more ambiguous real-world scenarios remains to be explored in subsequent research.

Third, the study adopted a strict definition of diagnostic correctness: only exact matches between the primary diagnosis and the reference standard were considered correct; broader superordinate labels and narrower subtype labels were scored as incorrect, and differential diagnoses were excluded from the primary outcome. This rigid binary criterion represents an important limitation because it may underestimate the broader clinical utility of both LLMs and physicians, particularly for responses that were “close to correct” or reasonably pointed toward broad disease categories. In real clinical practice, correctly identifying a broad rare disease category, narrowing the diagnostic range to a plausible disease family, or suggesting a closely related subtype may still be clinically meaningful because it may prompt referral to specialized centers, targeted genetic testing, additional imaging or laboratory evaluation, and earlier consideration of rare disease pathways. As a result, our strict exact-match approach may have underestimated diagnostic responses that were not fully correct but could still provide useful clinical direction. Concurrently, this study did not assign quantitative scores to the quality of differential diagnosis lists, thereby overlooking nuanced dimensions, such as the educational and suggestive value of high-quality differential diagnoses despite errors in the primary diagnosis. This represents an area for further improvement in future research. In addition, the diagnostic task requirements were not fully identical for the LLMs and physicians. The LLMs were explicitly prompted to generate 1 primary diagnosis and 5 differential diagnoses, whereas physicians were asked to provide only a single final primary diagnosis to reduce workload and maintain response quality. Although the primary outcome for both LLMs and physicians was evaluated using the same criterion—whether the primary diagnosis matched the reference standard—the process of generating a differential diagnosis list may have encouraged the LLMs to consider a broader diagnostic space and refine their primary diagnosis. In addition, prompting the LLMs to generate differential diagnoses may have acted as a chain-of-thought–like structured reasoning prompt, providing the models with an additional reasoning scaffold that was not available to physicians, who were asked to provide only a single final diagnosis. This potential cognitive advantage further limits the validity of the baseline LLM-versus-physician comparison. Therefore, the baseline comparison between standalone LLMs and physicians may have partly favored the LLMs. Future studies should use more symmetrical task designs, such as asking both LLMs and physicians to provide primary and differential diagnoses or evaluating both primary-diagnosis accuracy and differential-diagnosis quality.

Fourth, the LLM-related conclusions are restricted to 4 general-purpose models and the specific versions assessed using basic, single-turn, text-only prompts in Chinese. The 2-stage physician-assistance workflow used only 1 representative high-performing LLM; therefore, the observed assistance effect may be partly model-specific and should not be assumed to apply equally to all high-performing LLMs. More advanced prompting strategies, such as iterative questioning, structured diagnostic reasoning prompts, retrieval-augmented generation, or prompt optimization, were not examined. Multimodal inputs, including imaging findings, structured laboratory data, genetic information, or other clinical data, were also not incorporated. Therefore, the observed model performance may not represent the maximum achievable diagnostic utility of these LLMs in more realistic or optimized clinical decision-support settings. As LLMs evolve and multimodal capabilities expand, absolute and relative performance may change. Furthermore, although publicly available web interfaces with default settings reflect common real-world use, this approach limits experimental reproducibility. Public web interfaces may undergo silent model updates, interface-level prompt changes, and backend adjustments to generation parameters that are not visible to users. Because we did not use version-locked APIs with fixed parameters, such as temperature, top-p, or system prompts, exact replication of the benchmarking conditions may not be possible. Therefore, the LLM benchmarking results should be interpreted as time- and interface-specific observations rather than fully reproducible, version-controlled performance estimates. Potential data leakage or training-data contamination cannot be completely excluded. Although disease-specific labels and identifiers were removed from the standardized case descriptions, these cases were constructed based on the Chinese Rare Disease Catalog and clinical phenotype descriptions from the NRDRS. Because mainstream LLMs are trained on large-scale internet, scientific, and biomedical corpora, they may have previously encountered catalog information, registry-derived descriptions, or similar symptom clusters. Therefore, the high baseline accuracy of some LLMs may partly reflect prior exposure or memorization rather than purely de novo diagnostic reasoning. Future studies should use prospectively collected or locally held cases that are unlikely to have appeared in public training corpora.

Fifth, the study sample consisted of 42 intermediate and senior orthopedic physicians from a single country and a limited number of institutions; junior physicians and other specialties were excluded. Although this design better reflects the group of physicians mainly responsible for diagnosing and managing rare orthopedic conditions in clinical practice, it may introduce selection bias, and the sample size remains relatively small. Therefore, the findings may not be directly generalizable to physicians from other countries, health care systems, specialties, or training levels. In stratified analyses, statistical power remained limited. The study may also have been underpowered to detect the interaction between physician seniority group and LLM assistance stage. Therefore, the nonsignificant interaction (*P*=.10) should not be interpreted as evidence of equivalent benefit across physician groups; larger studies are needed to evaluate possible seniority-related differences in LLM-assisted benefit. Furthermore, the same set of 40 cases was used in the pre- and post-LLM–assisted diagnostic rounds, and no formal washout period or crossover design was implemented. To reduce additional information-seeking and external learning effects, both rounds were completed on the same day for each physician, and physicians were not allowed to consult textbooks, guidelines, online resources, or other external reference materials between the 2 rounds. This closed-book design created an artificially constrained physician baseline because clinicians typically use textbooks, guidelines, online databases, specialist resources, or other digital reference tools when evaluating suspected rare diseases. Thus, baseline physician performance should be interpreted as unaided, memory- and experience-based diagnosis, not as performance under standard reference-supported workflows. The observed benefit of LLM assistance therefore reflects its incremental value over a closed-book setting rather than over routine reference-supported clinical practice. In addition, potential learning effects and recall bias from repeated exposure to the same cases could not be fully separated from the effect of LLM assistance. In particular, the same-day reassessment design may have introduced short-term memory recall bias because physicians could have retained their initial impressions, baseline uncertainties, or memory of difficult cases when reviewing the LLM suggestions and completing the second round. Although the mixed-effects logistic regression model included random intercepts for physician identity, case identity, and the physician-by-case pair to account for paired dependency at the same physician-case level, residual correlation or model misspecification cannot be completely excluded in this simulated repeated-case design. Moreover, the analysis remained based on standardized simulated cases rather than real-world clinical encounters. Therefore, the observed pre-post improvement may overestimate the independent contribution of the LLM and should be interpreted as evidence from an exploratory proof-of-concept workflow evaluation rather than as a definitive estimate of the absolute diagnostic benefit of LLM assistance. Prospective validation in real clinical workflows, ideally using randomized, crossover, washout-period, or independent-case designs, is needed to better separate the effect of LLM assistance from recall, repeated-exposure, and learning effects. The mixed-effects logistic regression results should also be interpreted cautiously. Although both SPSS GENLINMIXED and R *glmmTMB* models showed a nonsignificant physician seniority group × LLM assistance stage interaction, the interaction *P* values differed (*P*=.10 vs *P*=.72). The large case-identity and physician-by-case random-effect variances suggest strong heterogeneity and possible model instability or near-complete separation, which may affect the precision of the interaction estimate. Therefore, the interaction result should not be interpreted as evidence of equivalent benefit across physician groups.

Sixth, although the clinicians who reviewed the LLM outputs were blinded to model identity, the evaluation of LLM outputs focused on primary-diagnosis correctness. In the second stage, physicians were provided with the representative LLM’s primary diagnosis, differential diagnoses, and brief rationale; however, the accuracy, completeness, and potential hallucination rate of the rationale were not independently assessed. Therefore, inaccurate or overly persuasive rationales may have influenced physicians’ post-LLM diagnostic decisions, either by supporting correct reasoning or by increasing the risk of automation bias. Future studies should separately evaluate the factual accuracy, hallucination rate, and clinical influence of LLM-generated rationales. Although the diagnostic transition analysis provided a more granular description of how diagnostic judgments changed after LLM assistance, the study did not directly record physicians’ confidence, reasoning processes, or reasons for accepting or rejecting LLM outputs. Therefore, potential automation bias and algorithmic aversion could only be inferred indirectly from transition patterns and should be examined more rigorously in future studies.

Finally, the questionnaire was developed for exploratory purposes, and although it was grounded in established frameworks, such as TAM and UTAUT, it was not subjected to full psychometric validation, such as factor analysis, construct validity testing, or test-retest reliability assessment. Moreover, the high Cronbach α observed across these diverse items may indicate overlapping item content or a generalized halo effect rather than validation of distinct constructs, such as trust, workload fit, safety, and perceived usefulness. Therefore, the questionnaire findings should be interpreted strictly as descriptive and exploratory indicators of physicians’ perceived usefulness, trust, safety, workload fit, and willingness to use the LLM-assisted workflow, rather than as definitive psychometric evidence of physician acceptance or psychological attitudes. The all-male physician cohort also represents another limitation, which may constrain the generalizability of the subjective questionnaire findings because perceptions of technology, trust in AI, and workflow acceptance may vary across demographic groups. Furthermore, although physicians reported favorable perceptions regarding workload and workflow fit, we did not collect objective time-tracking metrics. Therefore, we were unable to quantify the time required for baseline diagnosis or the additional time needed to read, interpret, and integrate the LLM’s outputs into the final diagnostic decision. As a result, the workflow-related findings should be interpreted as subjective perceptions rather than objective evidence of time efficiency. Future studies should incorporate objective workflow metrics, such as time to diagnosis and integration time, to further evaluate the practical feasibility of LLM-assisted diagnostic workflows in real clinical settings. These findings should be viewed as a foundation for designing more rigorously validated human–AI collaboration and implementation studies.

### Comparison With Previous Work

Previous studies have shown that LLMs can support medical diagnosis and decision-making by synthesizing clinical information and generating differential diagnoses or management suggestions [13-15,44]. In orthopedics, existing LLM studies have mainly addressed common conditions, including osteoarthritis diagnosis based on patient-reported questionnaires and knee or shoulder treatment decisions based on MRI reports [18,45]. Recent reviews and trend analyses further suggest that orthopedic LLM research remains concentrated on patient education, guideline-concordance checks, and decision support for common disorders, with limited empirical evidence for rare bone diseases or orthopedic-related rare diseases [18,22,46,47].

In the rare disease domain, only a few studies have started to explore LLM-based diagnosis. Zhong et al [5] evaluated LLM performance using the Chinese Rare Disease Catalog and showed the feasibility of catalog-driven rare disease benchmarking. Yu et al [17] assessed LLM-assisted diagnosis in rare hematologic diseases and suggested that LLM support may help narrow experience gaps among physicians. Shyr et al [16] evaluated LLMs using complex cases from the Undiagnosed Diseases Network and showed that LLMs could identify correct or closely related diagnoses among the top suggestions in some cases. However, that study did not target a specific specialty or systematically evaluate changes in clinicians’ diagnostic behavior or human–AI collaboration patterns. Building on these studies, our study extends the literature by focusing on an orthopedic-relevant rare disease subset, comparing multiple LLMs with intermediate and senior orthopedic physicians using the same case set, and evaluating a physician-in-the-loop LLM-assisted workflow with both objective diagnostic outcomes and subjective physician perceptions. Therefore, this study aimed to extend and complement existing research on LLM-assisted rare disease diagnosis by focusing on the diagnostic performance, physician-support effect, and subjective acceptance of LLMs in orthopedic-related rare diseases.

From the perspective of digital medicine and CDSSs, our findings suggest that, in a knowledge-sparse domain such as orthopedic-related rare diseases, high-performing LLMs showed strong primary-diagnosis performance under the present task setting and may serve as text-based knowledge-support tools for physicians. However, because the LLMs were prompted to generate both a primary diagnosis and differential diagnoses, whereas physicians provided only a single final primary diagnosis, direct comparisons between standalone LLMs and physicians should be interpreted with caution. In addition, the strict exact-match accuracy metric and the use of basic single-turn text prompts may have underestimated the broader clinical utility of both physicians and LLMs, particularly their ability to identify plausible disease categories, generate useful differential diagnoses, or guide subsequent referral and testing. At the individual level, all physicians benefited from LLM assistance; at the case level, LLM assistance reduced the number of cases for which intermediate and senior physicians were mostly incorrect and decreased the set of diseases solvable only by the LLM, implying that some rare disease knowledge may be “transferred” from the model to clinicians through human–AI interaction. After LLM assistance, some senior physicians with lower baseline accuracy showed numerically greater improvement, although this exploratory association was not statistically significant; meanwhile, some intermediate physicians achieved accuracy comparable to that of senior physicians. These descriptive findings raise the possibility that LLM assistance may help reduce group-level recognition gaps in rare disease diagnosis, although seniority-related differences in individual diagnostic correctness may persist. Overall, this study offers preliminary empirical evidence for the use of general-purpose LLMs as scalable knowledge resources and decision-support tools for diagnosing orthopedic-related rare diseases. It holds particular potential for application in health care settings that lack specialist resources for rare conditions. Future research may further explore integrating such models into clinical workflows for critical applications, including outpatient alerts, complex case discussions, and training assessments.

Our case-level analyses further revealed how LLMs may shape the distribution of case recognition across physicians and the model. LLM assistance increased the intersection of diseases correctly identified by the human groups and the model while reducing the gap in which only the LLM could make the correct call. In practice, this may translate into fewer “LLM-only hits” and more “shared understanding” between humans and the model. Concurrently, a small subset of cases remained difficult for both physicians and the model, highlighting the need for cautious integration and continuous monitoring of failure modes. These persistent postassistance blind-spot cases mainly involved metabolic bone diseases and a movement-disorder–related condition with overlapping or nonspecific musculoskeletal manifestations, suggesting that both physicians and LLMs may struggle when standardized text descriptions provide limited disease-specific distinguishing features.

By incorporating a brief questionnaire, the physicians’ subjective attitudes toward the LLM-assisted diagnostic workflow were also explored. The internal consistency was high (Cronbach α=0.902), and the scores were generally favorable, particularly regarding learning and educational value, perceived safety, workload and workflow fit, as well as willingness to recommend. However, given that the 8 items covered conceptually diverse dimensions, such as trust, explainability, workload fit, safety, and willingness to recommend, this high Cronbach α value may also reflect item redundancy or a generalized positive response tendency rather than true multidimensional construct validity. Therefore, the questionnaire results should be interpreted primarily as reflecting a generalized favorable sentiment toward the LLM-assisted workflow, rather than as validated evidence for each specific subdimension. Essentially, acceptance was similar among intermediate and senior physicians, and a clear pattern of more experienced clinicians being uniformly more skeptical was not observed. These findings provide exploratory, questionnaire-based evidence of perceived acceptability for similar human–AI collaborative workflows within multitiered orthopedic teams, but they should not be interpreted as definitive psychometric evidence of physician acceptance.

However, subjective acceptance and objective benefit showed weak correlations in this study. This discrepancy may reflect ceiling effects in questionnaire scores, limited sample size, or different determinants of subjective perception and diagnostic gain. Physicians’ attitudes may be shaped by risk perception, workload concerns, and general views toward LLMs, whereas actual diagnostic improvement may depend more on baseline rare disease knowledge and the ability to integrate LLM suggestions into case-specific reasoning. Therefore, even when LLM-assisted tools are perceived as useful and acceptable, targeted training, usage guidelines, and feedback mechanisms remain necessary to translate positive attitudes into stable diagnostic improvement.

China has developed its own National Rare Disease Catalog, focusing on diseases with relatively high burden, clear diagnostic criteria, and available treatment options in the local epidemiologic context. Although global databases such as Online Mendelian Inheritance in Man and Orphanet provide extensive rare disease information, geographic differences in prevalence and disease spectra may limit their direct use as clinical implementation frameworks. By constructing cases from the Chinese catalog and focusing on orthopedic-related rare diseases, this study highlights the need for region-specific LLM validation.

The use of Chinese as the interaction language also reflects real-world Chinese clinical settings and avoids translation-related term mismatches. However, because many LLMs are still trained predominantly on English-language corpora, future studies should evaluate rare disease diagnostic performance across multiple languages. Chimirri et al [48], using 10 languages and 4917 cases, similarly suggested the practical utility of LLMs in rare disease diagnosis across non-English clinical environments.

In our study, DeepSeek-V3.2 showed lower accuracy than the other 3 LLMs, which may reflect differences in training corpora, medical-domain fine-tuning, rare disease coverage, and Chinese-language adaptation. Because this study evaluated 4 LLMs within a specific orthopedic-related rare disease task and a single Chinese-language interaction setting, the observed differences may also reflect variations in cross-lingual knowledge integration and precise disease-name matching. Therefore, these findings should be interpreted as task- and time-specific rather than as general rankings of model capability across clinical scenarios or linguistic environments.

Although high-performing LLMs achieved high diagnostic accuracy and improved physician performance in this simulated workflow, they should remain knowledge-support tools rather than autonomous diagnostic systems. In rare disease settings, LLM outputs may be incomplete, outdated, or hallucinatory [49,50] and should be critically appraised by qualified clinicians using full clinical data, imaging, laboratory results, and current guidelines. Future implementation should proceed cautiously through secure systems, privacy safeguards, and prospective evaluation in real clinical workflows, ensuring that LLM-assisted diagnostic systems augment rather than replace existing diagnostic processes.

### Conclusions

In orthopedic-related rare diseases derived from the Chinese Rare Disease Catalog, advanced LLMs achieved high diagnostic accuracy. In the 2-stage workflow, LLM assistance was significantly associated with higher physician diagnostic correctness after accounting for physician-, case-, and physician-case–level clustering. Senior physicians retained higher overall diagnostic correctness than intermediate physicians, and the nonsignificant group-by-stage interaction should not be interpreted as evidence of equivalent benefit across experience levels. Secondary case-level analyses suggested attenuation of group-level gaps in case recognition patterns, and physicians reported broadly positive perceptions of the workflow. Given the same-day repeated-case design and potential short-term recall bias, these exploratory findings should be interpreted cautiously but support further prospective evaluation of LLMs as knowledge-support tools for rare disease diagnosis in orthopedics.

## Appendix

Multimedia Appendix 1 Catalog of Rare Orthopedic Diseases (Chinese monograph), names of 207 rare diseases listed in the Chinese Rare Disease Catalog, and schematic diagrams illustrating screening procedures for 40 orthopedic-related rare diseases.

Multimedia Appendix 2 Main features of the 4 large language models (LLMs) and runtime/prompt-control settings used in this study.

Multimedia Appendix 3 Baseline demographic and professional characteristics of participating orthopedic physicians.

Multimedia Appendix 4 Contents of the physician subjective questionnaire and score distribution across each item for intermediate and senior physicians.

Multimedia Appendix 5 Case questionnaire for 40 orthopedic-related rare diseases, available in both Chinese and English formats.

Multimedia Appendix 6 Evaluation results of the 4 large language models (LLMs) for 40 orthopedic-related rare diseases.

Multimedia Appendix 7 Evaluation results of intermediate and senior physicians for 40 orthopedic-related rare diseases.

Multimedia Appendix 8 Equivalent R generalized linear mixed-effects model results.

Multimedia Appendix 9 Supplementary analyses of physician-level diagnostic accuracy, diagnostic transition patterns, and physicians’ subjective evaluation before and after LLM assistance.

Multimedia Appendix 10 Shared human–large language model (LLM) blind-spot cases before and after LLM assistance.

## Abbreviations

CDSS: clinical decision support system
LLM: large language model
NRDRS: National Rare Disease Registry System
SCA: spinocerebellar ataxia
TAM: technology acceptance model
UTAUT: unified theory of acceptance and use of technology

## Data Availability

All data generated or analyzed during this study are included in this article and its Multimedia Appendices. Additional data supporting the findings of this study are available from the corresponding author upon reasonable request.
