# Supplementary material for: Diagnostic Performance of Large Language Models for Orthopedic-Related Rare Diseases and Their Impact on Physicians’ Diagnostic Accuracy: 2-Stage Comparative Evaluation Study Based on the Chinese Rare Disease Catalog
Source: J Med Internet Res. 2026 Jul 24;28:e92931. doi: 10.2196/92931 (PMC13451871; doi:10.2196/92931)
Supplement: Supplementary file 1 [file jmir_v28i1e92931_app10.docx]

**Multimedia Appendix 10.** Shared human-LLM blind-spot cases before and after LLM assistance.

| **Diagnosis** | **Before LLM assistance** | **After LLM assistance** | **Brief characterization** |
| --- | --- | --- | --- |
| Asphyxiating Thoracic Dystrophy (Jeune Syndrome) | Yes | No | Skeletal dysplasia with a small/narrow thorax, short ribs and limbs, abnormal pelvic morphology, respiratory insufficiency related to thoracic restriction, and possible renal, hepatic, retinal, and neurologic involvement. The complex multisystem skeletal phenotype may overlap with other skeletal dysplasias. |
| Hypophosphatemic Rickets | Yes | Yes | Metabolic bone disease with rickets-like skeletal deformities, including square skull, pigeon chest, beaded ribs, genu varum or valgum, growth retardation, short stature, waddling gait, progressive skeletal deformity, fractures, bone pain, dental abnormalities, and adult weakness or pathologic fractures. These manifestations may overlap with rickets, osteomalacia, and other metabolic bone disorders. |
| Primary Hereditary Dystonia | Yes | Yes | Movement-disorder-related condition characterized by dystonia, abnormal postures, and involuntary movements involving the head and neck, trunk, and limbs; symptoms may worsen with voluntary movement and improve during rest or sleep. Because the main presentation is neuromuscular rather than primary bone or joint disease, orthopedic clues may be indirect or nonspecific. |
| Tumor-Induced Osteomalacia | Yes | Yes | Metabolic/paraneoplastic bone disease presenting with nonspecific musculoskeletal features, including lower-limb, back, or intercostal bone pain, generalized weakness, mobility impairment, multiple pathologic fractures, spinal deformity or height loss, and tooth loosening or loss. These features may overlap with osteoporosis, osteomalacia, degenerative disease, or other fracture-related conditions. |
