# Supplementary material for: Diagnostic Performance of Large Language Models for Orthopedic-Related Rare Diseases and Their Impact on Physicians’ Diagnostic Accuracy: 2-Stage Comparative Evaluation Study Based on the Chinese Rare Disease Catalog
Source: J Med Internet Res. 2026 Jul 24;28:e92931. doi: 10.2196/92931 (PMC13451871; doi:10.2196/92931)
Supplement: Supplementary file 2 [file jmir_v28i1e92931_app2.docx]

**Multimedia Appendix 2.** Main features of the four LLMs and runtime/prompt-control settings used in this study.

**DeepSeek-V3.2**

DeepSeek-V3.2 (released December 1, 2025) is a long-context sparse-attention LLM that prioritizes training/inference efficiency while delivering GPT-5-level reasoning and strong agent/tool-use capability [1]. Its distinguishing features include DeepSeek Sparse Attention (DSA) for efficient long-context processing, a scaled RL post-training pipeline (with a high-compute V3.2-Speciale variant reported to exceed GPT-5 and approach Gemini-3.0-Pro reasoning), and a large-scale agentic task synthesis pipeline that improves instruction compliance and generalization in interactive tool environments. It further emphasizes controlled output length with lower compute cost, and is reported to achieve gold-medal-level performance in the 2025 IMO/IOI [2].

**ChatGPT-5.0 (*OpenAI*)**

ChatGPT-5.0 was launched in August 7th, 2025 as the successor to the GPT-4o and o1 series by OpenAI. The model integrates multi-agent reasoning modules and an adaptive self-evaluation mechanism that enables reflective learning and dynamic reasoning. It incorporates cross-modal capabilities (text, image, code, and audio) and introduces a reinforcement-of-reasoning (“RoR”) framework to enhance consistency and traceability of multi-step reasoning. ChatGPT-5.0 supports real-time web retrieval, personalized toolchains, and extended contextual comprehension beyond 1 million tokens. According to OpenAI, the model exhibits >95th percentile performance on MMLU, GSM8K, and HumanEval, outperforming GPT-4o by 15–20 points in long-form problem solving and medical-reasoning tasks. Its integration with API ecosystems (Docs, Browse, Canvas Tools) makes it particularly suitable for scientific and clinical applications requiring interpretability and data-safety [3].

**Claude Sonnet 4.5 (*Anthropic*)**

Claude Sonnet 4.5 was introduced by Anthropic in September 2025, marking the fourth-generation release of its “Sonnet” series. It integrates Anthropic’s Constitutional AI 2.0 framework, yielding significant advancements in ethical alignment, reliability, and high-stakes reasoning. Claude Sonnet 4.5 exhibits substantial gains in scientific reasoning, biomedical comprehension, and long-context coherence, with a context window up to 500 K tokens and cross-domain memory enhancement. Benchmark data published by Anthropic show that Claude Sonnet 4.5 outperforms Claude 3.5 Sonnet by approximately 30 % in complex document synthesis and 20 % in long-form mathematical proof tasks. Its expanded browser and file-tool integrations allow structured information retrieval and interpretation from scientific sources in real time [4].

**Gemini 2.5 Pro (*Google*)**

Gemini 2.5 Pro was launched in May 6th 2025 by Google DeepMind as the successor to Gemini 1.5 Pro. This iteration introduces multi-modal reasoning across text, code, image, and video, and supports 8-million-token context length, setting a new industry record. Gemini 2.5 Pro combines Transformer-Mixture-of-Experts architecture with an adaptive memory mechanism, enabling streamlined long-context processing and high-efficiency retrieval augmentation. DeepMind reports that Gemini 2.5 Pro achieves state-of-the-art scores in BigBench-Hard, MMLU, and ARC-Challenge, and exceeds Gemini 1.5 Pro by approximately 18 % in scientific reasoning and code-generation accuracy. Moreover, the model is optimized for real-time research workflow integration, including direct Google Workspace and Vertex AI connectivity, making it particularly suitable for academic and enterprise use [5]. In addition to being highly performant, the Gemini 2.5 models maintain strong safety standards and, compared to their 1.5 counterparts, are much more helpful. They are less likely to refuse to answer important user queries or respond with an overly sanctimonious tone. Gemini 2.5 exhibited notable increases in Critical Capabilities, including cybersecurity and machine learning R&D. However, the model has not crossed any Critical Capability Levels.

**References**

1. DeepSeek-V3.2: Efficient Reasoning & Agentic AI. Latest updated December 2025. Accessed December 1, 2025. [https://huggingface.co/deepseek-ai/DeepSeek-V3.2]

2. DeepSeek-AI. DeepSeek-V3.2-Exp: Boosting Long-Context Efficiency with DeepSeek Sparse Attention [Internet]. GitHub repository; 2025 [cited 2025 Dec 30]. [https://github.com/deepseek-ai/DeepSeek-V3.2-Exp]

3. OpenAI. Introducing GPT-5 and the Reasoning Core. Published October 2025. Accessed December 30, 2025. [https://openai.com/gpt-5/]

4. Anthropic. System Prompts [Internet]. Claude Docs (Anthropic). 2025 Nov 19 [cited 2025 Dec 30]. [ https://platform.claude.com/docs/en/release-notes/system-prompts#claude-sonnet-4-5]

5. Comanici G, Bieber E, Schaekermann M, Pasupat I, Sachdeva N, Dhillon I, et al. Gemini 2.5: Pushing the Frontier with Advanced Reasoning, Multimodality, Long Context, and Next Generation Agentic Capabilities. arXiv [cs.CL]. 2025; arXiv:2507.06261v6 (last revised 19 Dec 2025). [https://arxiv.org/html/2507.06261v6#S2]

**Table.** Runtime and prompt-control settings used in this study

| **Variable** | **Setting in this study** |
| --- | --- |
| Query operator | One investigator (ZM, 6 years of experience) |
| Evaluation period | Within a 20-day evaluation period |
| Access mode | Publicly available web interface of each model |
| Input language | Chinese |
| Prompt type | Single-turn diagnostic prompt |
| Case input | Standardized Chinese clinical vignette |
| Query repetition | Each case was queried once independently for each model |
| Session control | Separate single-turn sessions with truncated conversation history |
| Required output | One primary clinical diagnosis and five differential diagnoses |
| Response recording | All responses were recorded |
| Regeneration/manual editing | No manual editing was performed; the default web-interface settings were used |
| Generation settings | No generation parameters were manually adjusted |
