# Supplementary material for: Diagnostic Performance of Large Language Models for Orthopedic-Related Rare Diseases and Their Impact on Physicians’ Diagnostic Accuracy: 2-Stage Comparative Evaluation Study Based on the Chinese Rare Disease Catalog
Source: J Med Internet Res. 2026 Jul 24;28:e92931. doi: 10.2196/92931 (PMC13451871; doi:10.2196/92931)
Supplement: Supplementary file 3 [file jmir_v28i1e92931_app3.docx]

**Multimedia Appendix 3.** Baseline demographic and professional characteristics of participating orthopedic physicians.

| **Characteristic** | **Intermediate physicians**  **(n=27)** | **Senior physicians**  **(n=15)** | **Total**  **(N=42)** |
| --- | --- | --- | --- |
| **Professional title, n (%)** |  |  |  |
| Attending physician | 27 (100.0) | — | 27 (64.3) |
| Associate chief physician | — | 9 (60.0) | 9 (21.4) |
| Chief physician | — | 6 (40.0) | 6 (14.3) |
| Years in orthopedic practice, mean (SD) | 11.74 (1.68) | 21.87 (3.70) | 15.36 (5.53) |
| Years in orthopedic practice, median (IQR) | 12 (10, 13) | 21.0 (19, 25) | 13 (11, 20) |
| **Gender, n (%)** |  |  |  |
| Male | 27 (100.0) | 15 (100.0) | 42 (100.0) |
| Female | 0 (0.00) | 0 (0.00) | 0 (0.00) |
| **Orthopedic subspecialty focus, n (%)** |  |  |  |
| Spine surgery | 10 (37.0) | 6 (40.0) | 16 (38.1) |
| Joint surgery | 5 (18.5) | 3 (20.0) | 8 (19.0) |
| Trauma orthopedics | 3 (11.1) | 1 (6.7) | 4 (9.5) |
| Sports medicine | 3 (11.1) | 2 (13.3) | 5 (11.9) |
| Pediatric orthopedics | 2 (7.4) | 1 (6.7) | 3 (7.1) |
| Bone tumor / musculoskeletal oncology | 4 (14.8) | 2 (13.3) | 6 (14.3) |
| **Practice setting, n (%)** |  |  |  |
| Specialized orthopedic center | 5 (18.5) | 2 (13.3) | 7 (16.7) |
| General hospital orthopedic department | 22 (81.5) | 13 (86.7) | 35 (83.3) |

Abbreviations: IQR, interquartile range; SD, standard deviation.
