# Supplementary material for: Diagnostic Performance of Large Language Models for Orthopedic-Related Rare Diseases and Their Impact on Physicians’ Diagnostic Accuracy: 2-Stage Comparative Evaluation Study Based on the Chinese Rare Disease Catalog
Source: J Med Internet Res. 2026 Jul 24;28:e92931. doi: 10.2196/92931 (PMC13451871; doi:10.2196/92931)
Supplement: Supplementary file 4 [file jmir_v28i1e92931_app8.docx]

**Multimedia Appendix 8.** Equivalent R generalized linear mixed-effects model results.

**A. Fixed effects from the equivalent R model**

| **Parameter** | **Estimate** | **Standard errors** | **Z value** | ***P* value** |
| --- | --- | --- | --- | --- |
| Physician seniority group | 16.347 | 1.859 | 8.794 | <.001 |
| LLM assistance stage | 14.723 | 0.905 | 16.275 | <.001 |
| Physician seniority group × LLM assistance stage | 0.419 | 1.167 | 0.359 | .72 |

**B. Random effects from the equivalent R model**

| **Random effect** | **Variance estimate** | **Standard errors** |
| --- | --- | --- |
| Physician identity random intercept | 7.908 | 4.242 |
| Case identity random intercept | 516.644 | 159.534 |
| Physician-by-case pair random intercept | 343.769 | 72.585 |
