# Supplementary material for: Diagnostic Performance of Large Language Models for Orthopedic-Related Rare Diseases and Their Impact on Physicians’ Diagnostic Accuracy: 2-Stage Comparative Evaluation Study Based on the Chinese Rare Disease Catalog
Source: J Med Internet Res. 2026 Jul 24;28:e92931. doi: 10.2196/92931 (PMC13451871; doi:10.2196/92931)
Supplement: Supplementary file 5 [file jmir_v28i1e92931_app1.zip › Multimedia Appendix 1/Catalogue of Rare Orthopaedic Diseases.pdf]

Rare orthopaedics diseases

Contents

Chapter 1: Abnormal development of the skeletal muscle system

Section 1: Chondrodysplasia punctata

(1) Non-rhizomelic chondrodysplasia punctata

(2) Rhizomelic chondrodysplasia punctata

(3) Astley-Kendall dysplasia

(4) Congenital hemidysplasia with ichthyosiform erythroderma and limb defects

(5) Greenberg dysplasia

(6) Keutel syndrome

Section 2: Acrodysostosis

Section 3: Diaphyseal dysplasia

Section 4: Metaphyseal chondrodysplasia

Section 5: Dysplasia epiphysealis hemimelica

Section 6: Multiple epiphyseal dysplasia

Section 7: Spondyloepiphyseal dysplasia

Section 8: Legg-Calve-Perthes disease

Section 9: Pseudoachondroplasia

Section 10: Osteogenesis imperfecta

Section 11: Pycnodysostosis

Section 12: Melorheostosis

Section 13: Osteopetrosis

Section 14: Klippel-Feil syndrome

Section 15: Congenital scoliosis

Section 16: Sprengel deformity

Section 17: Arthrogryposis Multiplex Congenita

Chapter 2: Metabolic and endocrine diseases

Section 1: Acromegaly

Section 2: Vitamin D deficiency rickets

Section 3: Hypophosphatemic rickets

Section 4: Hypophosphatasia

Section 5: Mucopolysaccharidosis

Section 6: Mucolipidosis

Section 7: Glycogen storage disease

Chapter 3: Muscle diseases

Section 1: Congenital muscular dystrophy

Section 2: Duchenne muscular dystrophy

Section 3: Emery-Dreifuss muscular dystrophy

Section 4: Facioscapulohumeral muscular dystrophy

Section 5: Limb-girdle muscular dystrophy

Section 6: Schwartz-Jampel syndrome

Chapter 4: Neurological diseases

Section 1: Hereditary spastic paraplegia

Section 2: Multifocal motor neuropathy

Section 3: Spinocerebellar ataxia

Section 4: Amyotrophic lateral sclerosis

Section 5: Spinal and bulbar muscular atrophy

Section 6: Spinal muscular atrophy

Section 7: Charcot-Marie-Tooth disease

Section 8: Multiple sclerosis

Section 9: Juvenile muscular atrophy of distal upper extremity

Chapter 5: Connective tissue diseases

Section 1: Marfan syndrome

Section 2: Stickler syndrome

Section 3: Meier-Gorlin syndrome

Section 4: Fibro-dysplasia ossificans progressiva

Chapter 6: Tumors and tumor-like diseases

Section 1: Desmoid tumor

Section 2: Hereditary multiple osteochondroma

Section 3: Maffucci syndrome

Section 4: Mesenchymal chondrosarcoma

Section 5: Paget disease of the bone

Section 6: Neurofibromatosis

(1) Neurofibromatosis type 1

(2) Neurofibromatosis type 2

(3) Schwannomatosis

Section 7: Erdheim-Chester disease

Chapter 7: Immune-related diseases

Section 1: Genetic immune dysregulatory syndromes (COPA syndrome)

Section 2: SAPHO syndrome

Section 3: Relapsing polychondritis

Section 4: Parsonage-Turner syndrome

Chapter 8: Infectious diseases

Section 1: Bone hydatid disease

Section 2: Muscle cysticercosis

Section 3: Bone syphilis

Chapter 9: Blood system diseases

Section 1: Multiple myeloma

Section 2: Fanconi anemia

Section 3: Langerhans cell histiocytosis

Chapter 10: Syndrome with partial multiple system dysplasia and skeletal deformity

Section 1: Acrocephalosyndactyly

Section 2: Hereditary osteodysplasia with acroostedolysis

Section 3: Holt-Oram syndrome

Section 4: Larsen syndrome

Section 5: McCune- Albright syndrome

Section 6: Weill-Marchesani syndrome

Section 7: Poland syndrome

Section 8: Proteus syndrome

Section 9: Schimke immunoosseous dysplasia

Section 10: Russell-Silver syndrome
