# Supplementary material for: Diagnostic Performance of Large Language Models for Orthopedic-Related Rare Diseases and Their Impact on Physicians’ Diagnostic Accuracy: 2-Stage Comparative Evaluation Study Based on the Chinese Rare Disease Catalog
Source: J Med Internet Res. 2026 Jul 24;28:e92931. doi: 10.2196/92931 (PMC13451871; doi:10.2196/92931)
Supplement: Supplementary file 8 [file jmir_v28i1e92931_app9.zip › Multimedia Appendix 9/Table S1.docx]

**Table S1.** Diagnostic performance of physicians before and after LLM assistance, and a comparison between the two phases.

|  | **Median (range), n (%)** | **Average accuracy rate** | **Before vs. after LLM assistance** |
| --- | --- | --- | --- |
| Intermediate physician before LLM assistance | 17 (12-24) [42.5 (30.0-60.0)] | 42.22% | *P* < .001 |
| Intermediate physician after LLM assistance | 27 (22-35) [67.5 (55.0-87.5)] | 68.80% |  |
| Senior physician before LLM assistance | 24 (17-27) [60.0 (42.5-67.5)] | 58.67% | *P* < .001 |
| Senior physician after LLM assistance | 34 (29-38) [85.0 (72.5-95.0)] | 83.33% |  |
