# Supplementary material for: Diagnostic Performance of Large Language Models for Orthopedic-Related Rare Diseases and Their Impact on Physicians’ Diagnostic Accuracy: 2-Stage Comparative Evaluation Study Based on the Chinese Rare Disease Catalog
Source: J Med Internet Res. 2026 Jul 24;28:e92931. doi: 10.2196/92931 (PMC13451871; doi:10.2196/92931)
Supplement: Supplementary file 8 [file jmir_v28i1e92931_app9.zip › Multimedia Appendix 9/Table S2.docx]

**Table S2.** Diagnostic performance of intermediate physicians before and after the LLM assistance.

| **ID** | **Before LLM-assisted intermediate physicians** | | **After LLM-assisted intermediate physicians** | |
| --- | --- | --- | --- | --- |
|  | **Frequency** | **Accuracy rate** | **Frequency** | **Accuracy rate** |
| No.1 | 14 | 35.0% | 25 | 62.5% |
| No.2 | 16 | 40.0% | 26 | 65.0% |
| No.3 | 17 | 42.5% | 26 | 65.0% |
| No.4 | 17 | 42.5% | 28 | 70.0% |
| No.5 | 15 | 37.5% | 27 | 67.5% |
| No.6 | 15 | 37.5% | 26 | 65.0% |
| No.7 | 18 | 45.0% | 27 | 67.5% |
| No.8 | 24 | 60.0% | 32 | 80.0% |
| No.9 | 14 | 35.0% | 23 | 57.5% |
| No.10 | 14 | 35.0% | 25 | 62.5% |
| No.11 | 12 | 30.0% | 22 | 55.0% |
| No.12 | 15 | 37.5% | 26 | 65.0% |
| No.13 | 13 | 32.5% | 23 | 57.5% |
| No.14 | 18 | 45.0% | 25 | 62.5% |
| No.15 | 21 | 52.5% | 35 | 87.5% |
| No.16 | 17 | 42.5% | 28 | 70.0% |
| No.17 | 18 | 45.0% | 28 | 70.0% |
| No.18 | 18 | 45.0% | 27 | 67.5% |
| No.19 | 17 | 42.5% | 27 | 67.5% |
| No.20 | 17 | 42.5% | 28 | 70.0% |
| No.21 | 16 | 40.0% | 28 | 70.0% |
| No.22 | 23 | 57.5% | 35 | 87.5% |
| No.23 | 13 | 32.5% | 25 | 62.5% |
| No.24 | 20 | 50.0% | 31 | 77.5% |
| No.25 | 16 | 40.0% | 28 | 70.0% |
| No.26 | 15 | 37.5% | 27 | 67.5% |
| No.27 | 23 | 57.5% | 35 | 87.5% |
