# Supplementary material for: Diagnostic Performance of Large Language Models for Orthopedic-Related Rare Diseases and Their Impact on Physicians’ Diagnostic Accuracy: 2-Stage Comparative Evaluation Study Based on the Chinese Rare Disease Catalog
Source: J Med Internet Res. 2026 Jul 24;28:e92931. doi: 10.2196/92931 (PMC13451871; doi:10.2196/92931)
Supplement: Supplementary file 8 [file jmir_v28i1e92931_app9.zip › Multimedia Appendix 9/Table S3.docx]

**Table S3.** Diagnostic performance of senior physicians before and after the LLM assistance.

| **ID** | **Before LLM-assisted senior physicians** | | **After LLM-assisted senior physicians** | |
| --- | --- | --- | --- | --- |
|  | **Accuracy frequency** | **Accuracy rate** | **Accuracy frequency** | **Accuracy rate** |
| No.1 | 25 | 62.5% | 34 | 85.0% |
| No.2 | 26 | 65.0% | 35 | 87.5% |
| No.3 | 26 | 65.0% | 33 | 82.5% |
| No.4 | 23 | 57.5% | 30 | 75.0% |
| No.5 | 27 | 67.5% | 35 | 87.5% |
| No.6 | 21 | 52.5% | 32 | 80.0% |
| No.7 | 21 | 52.5% | 31 | 77.5% |
| No.8 | 25 | 62.5% | 36 | 90.0% |
| No.9 | 24 | 60.0% | 34 | 85.0% |
| No.10 | 23 | 57.5% | 30 | 75.0% |
| No.11 | 25 | 62.5% | 35 | 87.5% |
| No.12 | 17 | 42.5% | 29 | 72.5% |
| No.13 | 21 | 52.5% | 35 | 87.5% |
| No.14 | 23 | 57.5% | 33 | 82.5% |
| No.15 | 25 | 62.5% | 38 | 95.0% |
