# Supplementary material for: Diagnostic Performance of Large Language Models for Orthopedic-Related Rare Diseases and Their Impact on Physicians’ Diagnostic Accuracy: 2-Stage Comparative Evaluation Study Based on the Chinese Rare Disease Catalog
Source: J Med Internet Res. 2026 Jul 24;28:e92931. doi: 10.2196/92931 (PMC13451871; doi:10.2196/92931)
Supplement: Supplementary file 8 [file jmir_v28i1e92931_app9.zip › Multimedia Appendix 9/Table S4.docx]

**Table S4.** Diagnostic transition patterns before and after LLM assistance.

| **Diagnostic transition pattern** | **Intermediate physicians, n (%)** | **Senior physicians, n (%)** | **Overall, n (%)** |
| --- | --- | --- | --- |
| Accurate before and after assistance | 456/1080 (42.2%) | 352/600 (58.7%) | 808/1680 (48.1%) |
| Changed from accurate to inaccurate | 0/1080 (0.0%) | 0/600 (0.0%) | 0/1680 (0.0%) |
| Changed from inaccurate to accurate | 287/1080 (26.6%) | 148/600 (24.7%) | 435/1680 (25.9%) |
| Inaccurate before and after assistance | 337/1080 (31.2%) | 100/600 (16.7%) | 437/1680 (26.0%) |

**Note:** Diagnostic transition patterns were calculated at the physician-case pair level. “Accurate before and after assistance” indicates an accurate diagnosis both before and after LLM assistance; “changed from accurate to inaccurate” indicates an accurate diagnosis before assistance but an inaccurate diagnosis after assistance; “changed from inaccurate to accurate” indicates an inaccurate diagnosis before assistance but an accurate diagnosis after assistance; and “inaccurate before and after assistance” indicates an inaccurate diagnosis both before and after assistance.
