# Supplementary material for: Diagnostic Performance of Large Language Models for Orthopedic-Related Rare Diseases and Their Impact on Physicians’ Diagnostic Accuracy: 2-Stage Comparative Evaluation Study Based on the Chinese Rare Disease Catalog
Source: J Med Internet Res. 2026 Jul 24;28:e92931. doi: 10.2196/92931 (PMC13451871; doi:10.2196/92931)
Supplement: Supplementary file 8 [file jmir_v28i1e92931_app9.zip › Multimedia Appendix 9/Table S5.docx]

**Table S5.** Descriptive statistics for the 8-dimensional scores of the physician subjective questionnaire and comparisons among physicians.

| **Items** | **Dimensions** | **Mean ± SD** | **Median (P25, P75)** | **Physician group comparison *P*-value** |
| --- | --- | --- | --- | --- |
| Q1 | Perceived usefulness | 4.17 ± 0.49 | 4 (4, 4) | .35 |
| Q2 | Trust level | 4.21 ± 0.56 | 4 (4, 5) | .28 |
| Q3 | Explainability | 4.21 ± 0.56 | 4 (4, 5) | .74 |
| Q4 | Learning/Educational value | 4.31 ± 0.56 | 4 (4, 5) | .78 |
| Q5 | Workload and workflow fit | 4.24 ± 0.53 | 4 (4, 5) | .63 |
| Q6 | Perceived safety | 4.26 ± 0.50 | 4 (4, 5) | .51 |
| Q7 | Intention to use in the future | 4.17 ± 0.54 | 4 (4, 4.25) | .76 |
| Q8 | Willingness to recommend | 4.24 ± 0.48 | 4 (4, 5) | .11 |
